# Dicarboxyl-terminated iron(ii) clathrochelates as ICD-reporters for globular proteins†

**DOI:** 10.1039/c9ra04102h

**Published:** 2019-08-05

**Authors:** Vladyslava Kovalska, Serhii Vakarov, Mykhaylo Losytskyy, Marina Kuperman, Nina Chornenka, Yuliya Toporivska, Elzbieta Gumienna-Kontecka, Yan Voloshin, Oleg Varzatskii, Andriy Mokhir

**Affiliations:** Institute of Molecular Biology and Genetics, NASU 150 Zabolotnogo St. 03143 Kyiv Ukraine v.kovalska@gmail.com; Princeton Biomolecular Research Labs 26A Saperne Pole St. 01042 Kyiv Ukraine; V.I. Vernadsky Institute of General and Inorganic Chemistry, NASU 32/34 Palladin Av. 03142 Kyiv Ukraine; Faculty of Chemistry, University of Wroclaw 14 F. Joliot-Curie St. 50-383 Wroclaw Poland; Nesmeyanov Institute of Organoelement Compounds RAS 28 Vavilova St. 119991 Moscow Russia; Kurnakov Institute of General and Inorganic Chemistry of the Russian Academy of Sciences 31 Leninsky Prosp. 119991 Moscow Russia; Organic Chemistry II, Friedrich-Alexander-University of Erlangen-Nuremberg Henkestr. 42 91054 Erlangen Germany

## Abstract

Cage metal complexes iron(ii) clathrochelates, which are inherently CD silent, were discovered to demonstrate intensive output in induced circular dichroism (ICD) spectra upon their assembly to albumins. With the aim to design clathrochelates as protein-sensitive CD reporters, the approach for the functionalization of one chelate α-dioximate fragment of the clathrochelate framework with two non-equivalent substituents was developed, and constitutional isomers of clathrochelate with two non-equivalent carboxyphenylsulfide groups were synthesized. The interaction of designed iron(ii) clathrochelates and their symmetric homologues with globular proteins (serum albumins, lysozyme, β-lactoglobulin (BLG), trypsin, insulin) was studied by protein fluorescence quenching and CD techniques. A highly-intensive ICD output of the clathrochelates was observed upon their association with albumins and BLG. It was shown that in the presence of BLG, different clathrochelate isomers gave spectra of inverted signs, indicating the stabilization of opposite configurations (*Λ* or *Δ*) of the clathrochelate framework in the assembly with this protein. So, we suggest that the isomerism of the terminal carboxy group determined preferable configurations of the clathrochelate framework for the fixation in the protein binding site. MALDI TOF results show the formation of BLG–clathrochelate complex with ratio 1 : 1. Based on the docking simulations, the binding of the clathrochelate molecule (all isomers) to the main BLG binding site (calyx) in its open conformation is suggested. The above results point that the variation of the ribbed substituents at the clathrochelate framework is an effective tool to achieve the specificity of clathrochelate ICD reporting properties to the target protein.

## Abstract 2

Method of asymmetric mono-ribbed functionalization of iron(ii) clathrochelates is developed, new compounds are studied as ICD reporters for globular proteins.

## Graphical abstract

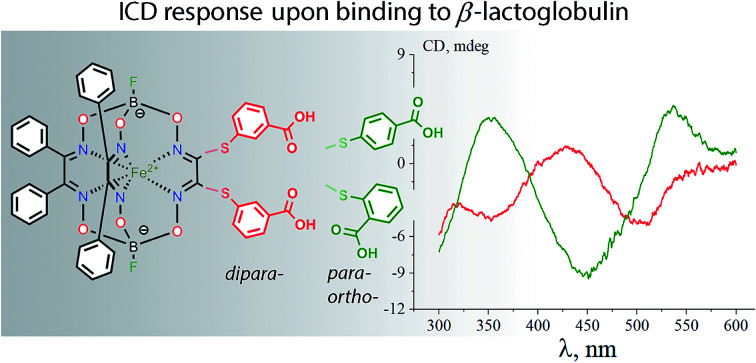

## 1. Introduction

The design and synthesis of macrocyclic compounds which are able to recognize specific surface elements of proteins seem to be undoubtedly important for various biochemical and biomedical applications.^1^ These surface elements are generally hardly recognizable by regular small organic molecules, because protein macromolecules commonly possess the large surface motifs without well-formed binding pockets and the low-molecular probes are poorly matched to them. Because protein macromolecules commonly possess large surface motifs without well-formed binding pockets, they are generally hardly recognizable by regular, low-molecular organic molecules/probes, which are poorly matched. A surface mimetic approach relays on a recognition of large areas of proteins' surfaces using functionalized supramolecular probes, which are able to form multiple non-covalent contacts, thus achieving strong and selective binding.^2,3^ The supramolecular agents possessing the above features are promising for use in advanced therapeutic approaches; targeting protein–protein interactions (PPIs), immobilization techniques, as well as the construction of sensors, affinity tags and protein-based materials are among the most prominent applications.^1^

Nowadays, several classes of molecules, such as crown ethers, pillarenes, porphyrins, cucurbiturils, cyclodextrins and calixarenes, as well as molecular tweezers, are reported^2–4^ to be able to recognize either the short peptides, or the molecular elements on protein surfaces. Similarly to PPIs, such a binding in aqueous solution is mainly driven by the hydrophobic effects, while other non-covalent interactions, such as electrostatic interactions and hydrogen bonding, typically play a secondary, modulatory role.^1^

Advances in supramolecular chemistry and structure-based drug design suggest transition metal complexes as another, important class of compounds efficiently targeting and non-covalently interacting with proteins.^5–8^ Strong and selective binding of a designed metallocomplex to a target protein requires a close match between the shape of a guest molecule, its charge distribution and hydrogen bonding ability and both the spatial and electronic structure of the binding sites of a protein.^6^ Metal complexes combine the flexibility in ligand design with an access to the variety of the coordination geometries, geometrical and optical isomers, and they are suited for optimisation of their abilities to form non-covalent interactions. Among others, macrobicyclic metal complexes, *i.e.* iron(ii) clathrochelates^9,10^ are three-dimensional, easy-to-modify molecular scaffolds prospective for the design of biologically active compounds. Their interactions with biomolecules have been extensively studied to date,^11–17^ revealing iron(ii) mono- and bis-clathrochelates to be efficient (submicromolar) inhibitors in the transcription system of T7 RNA polymerase,^11^ and (low micromolar) Taq DNA polymerase.^12^ The *in silico* simulated mode of inhibition of T7 RNA polymerase activity by these cage complexes suggests an inclusion of the inhibitor's clathrochelate molecule into a binding pocket which is formed by the molecules of polymerase, matrix DNA and o synthesized RNA (*i.e.*, an inhibition of the protein–nucleic acids interactions). Highest *in vitro* inhibitory activity in the transcription systems of these polymerases has been observed for cage iron(ii) complexes with functionalizing ribbed carboxyphenylsulfide substituents in an α-dioximate chelate fragment of the macrobicyclic frameworks. The same carboxyphenylsulfide iron(ii) mono- and bis-clathrochelates are also reported^13,14^ to be able to affect the protein–protein interactions in amyloid self-aggregation. It was shown, that both the kinetics of fibril formation and the morphology of protein fibrils formed by insulin and lysozyme, were affected by the presence of these macropolycyclic complexes.^13,14^

Recently, the formation of supramolecular complexes between iron(ii) clathrochelates and native serum albumins has been observed.^15^ An ability of inherently CD silent cage molecules to give an induced circular dichroism (ICD) output in the visible range upon such an assembling has been reported.^16^ The ICD response was explained^6^ by the existence of the free clathrochelate framework as an equilibrium of enantiomers with trigonal prismatically (TP)-distorted and trigonal antiprismatically (TAP)-distorted optically active conformations (*Δ*- and *Λ*-conformations, respectively). Binding of the clathrochelate to an asymmetric binding site of the protein leads to selective fixation (and thus accumulation) of one of these enantiomers. Intensive ICD signals observed for iron(ii) clathrochelates with ribbed carboxyl-terminated phenylsulfide substituents were shown affected by a constitutional isomerism of the substituents (*i.e.*, *ortho*-, *meta*- or *para*-positions of the terminal carboxyl groups).^16^ This behaviour underlines the importance of the precise electrostatic (polar) interactions between the terminal carboxyl groups and the positively charged amino acid residues of a protein for the fixation of a given conformation of the clathrochelate framework. An ability of the hexacarboxyphenylsulfide iron(ii) cage complexes to discriminate between the proteins of similar structures (*i.e.*, human and bovine serum albumins) by the ICD outputs has been recently reported.^17^

**Scheme 1:**
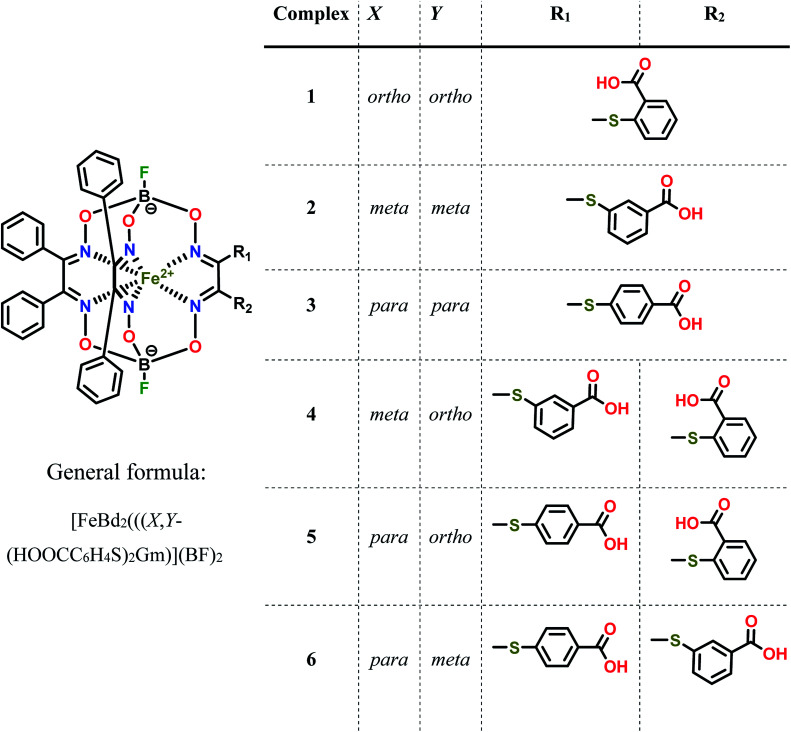
Chemical drawings of the isomeric dicarboxyl-terminated iron(ii) cage complexes and their dichloroclathrochelate precursor.

**Fig. 1:**
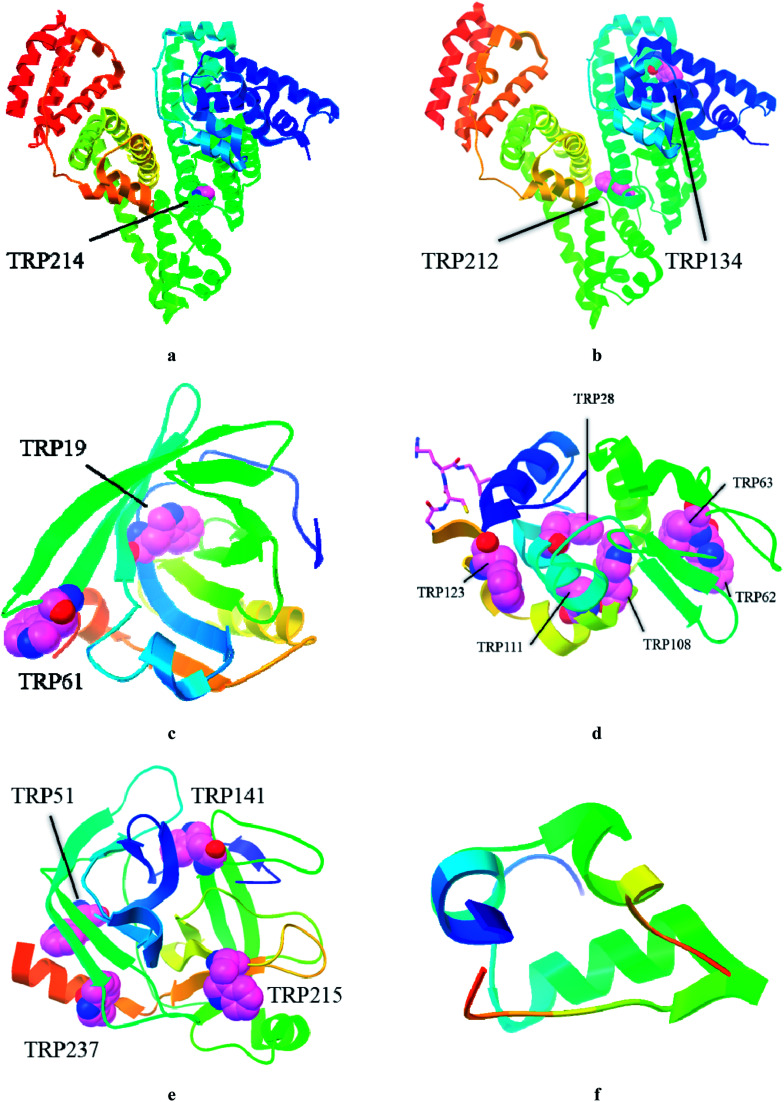
Structures of studied globular proteins taken from RSCB protein data bank: HSA (PDB ID: 4K2C, (a), BSA (PDB ID: 4JK4, (b), BLG (PDB ID: 2BLG, (c), LYZ (PDB ID: 1DPX, (d), TPS (PDB ID: 1FNI, (e), INS (PDB ID: 5BTS, (f).

To further explore clathrochelates as prospective ICD reporters, which are sensitive to the structure of a protein, we have synthesized novel monoribbed-functionalized iron(ii) cage complexes bearing two non-equivalent ribbed carboxylphenylsulfide groups. For this, the synthetic approach allowing modification of one chelate α-dioximate fragment of a macrobicyclic framework with two non-equivalent ribbed substituents was developed. The interactions between a complete set of the isomeric clathrochelates with two equivalent or non-equivalent carboxyphenylsulfide groups (Scheme 1) and a series of globular proteins, *i.e.* bovine and human serum albumins (BSA and HSA, respectively), lysozyme (LYZ), beta-lactoglobulin (BLG), trypsin (TPS) and insulin (INS) (Fig. 1) were characterized. The assembling was studied by two complimentary spectral methods, the protein fluorescence quenching and CD spectroscopy. In addition, the formation of the BLG–clathrochelate assemblage was experimentally confirmed by MALDI-TOF mass spectrometry data. The molecular docking calculations were used to deduce the geometry of this complex.

## 2. Experimental

### 2.1. Materials

The reagents used, FeCl_2_·4H_2_O, α-benzildioxime (H_2_Bd), BF_3_·O(C_2_H_5_)_2_, *ortho*-, *meta*- and *para*-mercaptobenzoic acids, sorbents, organic bases and solvents were obtained commercially (SAF®). Dichloroglyoxime was obtained by a known procedure.^18^ The dichloroclathrochelate precursor FeBd_2_(Cl_2_Gm)(BF)_2_ was prepared as described elsewhere.^19^ The dicarboxyphenylsulfide iron(ii) clathrochelates with equivalent functionalizing ribbed substituents were synthesized as described elsewhere.^20^ BSA and HSA were obtained commercially (SAF® and Fisher Bioreagents, respectively). BLG, LYZ, human insulin, trypsin and DMSO of an analytical grade were also purchased from SAF®. 50 mM Tris–HCl buffer (pH 7.9) was used as a solvent in all the spectral studies.

### 2.2. Synthesis, analytical and spectral characteristics of the iron(ii) clathrochelates 4–6

The following general *one-pot* procedure was used for the preparation of the heterodifunctionalized iron(ii) clathrochelates FeBd_2_((X-HOOCC_6_H_4_S)(Y-HOOCC_6_H_4_S)Gm)(BF)_2_ (where X and Y are *ortho*-, *meta*- or *para*-substituent) with non-equivalent carboxyl-terminated ribbed substituents.

Complex FeBd_2_(Cl_2_Gm)(BF)_2_ (0.75 g, 1 mmol) was dissolved/suspended in dichloromethane (100 ml) at 0 °C and the corresponding *ortho*-, *meta*- or *para*-mercaptobenzoic acid (0.154 g, 1 mmol) and triethylamine (0.35 ml, 2.5 mmol) were added. The reaction mixture was stirred for 12 h at r.t., then elute of this mixture was washed with water (100 ml), diluted (2%) aqueous hydrochloric acid (100 ml) and dried with Na_2_SO_4_. The dichloromethane solution was flash-chromatographically separated on silica gel (10 mm layer, eluents: dichloromethane to elute the parent dichloroclathrochelate and, then, dichloromethane–ethyl acetate 9 : 1 mixture to obtain the target product). The first elute was discarded and the second elute was collected, evaporated to dryness and dissolved in DMSO (5 ml). Then, another constitutional isomer of mercaptobenzoic acid (0.2 g, 1.35 mmol) and triethylamine (0.49 ml, 3.5 mmol) were added to this DMSO solution at r.t. The reaction mixture was stirred for 1 h and poured into a diluted (2%) aqueous hydrochloric acid (50 ml). The precipitate formed was filtered off, washed with water and dried *in air*. The product was extracted with dichloromethane and this extract was dried with Na_2_SO_4_. The obtained solution was evaporated to a small volume (approximately 10 ml) and precipitated with hexane (50 ml). The precipitate was filtered off, washed with hexane and dried *in vacuo*.

#### 2.2.1 FeBd_2_(*meta*,*ortho*-(HOOCC_6_H_4_S)_2_Gm)(BF)_2_ (4)

Yield: 53%. Calcd for C_44_H_30_N_6_B_2_F_2_FeO_10_S_2_·0.5H_2_O: C, 53.31; H, 3.15; N, 8.48. Found (%): C, 53.20; H, 3.25; N, 8.63. ESI-LC/MS (acetonitrile): *m*/*z*: 965 [M without hydroxygroup]^+^, 982 [M]^+^˙ ^1^H NMR (DMSO, *δ*, ppm): 6.65 (d, *J* = 7.7 Hz, 1H), 7.26 (t, 1H, *meta*-5-Ph), 7.35 (d, *J* = 3.3 Hz, 20H), 7.48 (s, 1H, *meta*-2-Ph), 7.56 (d, 6.6 Hz, 1H), 7.69 (d, *J* = 7.6 Hz, 1H), 7.88 (d, *J* = 6.6 Hz, 1H). ^13^C{^1^H} NMR (DMSO, *δ*, ppm): 126.24, 126.69, 127.99, 128.09, 128.40, 128.69, 128.82, 129.20, 129.66, 130.33, 130.48, 131.22, 131.89, 132.10, 132.29, 133.45, 134.89 (all s, Ar + Ph), 148.06, 149.35 (two s, SC=N), 157.41, 157.58 (two s, PhC=N), 166.39, 167.72 (two s, COOH). UV-Vis (CH_3_OH) *λ*_max_, nm (*ε* × 10^−3^, L mol^−1^ cm^−1^) 248(31), 292(16), 368(5), 430(5), 460(8), 487(12), 511(11).

#### 2.2.2 FeBd_2_(*para*,*ortho*-(HOOCC_6_H_4_S)_2_Gm)(BF)_2_ (5)

Yield: 60%. Calcd for C_44_H_30_N_6_B_2_F_2_FeO_10_S_2_·0.5H_2_O: C, 53.31; H, 3.15; N, 8.48. Found (%): C, 53.23; H, 3.27; N, 8.25. ESI-LC/MS (toluene): *m*/*z*: 982 [M]^+ 1^H NMR (CD_2_Cl_2_, *δ*, ppm): 6.77 (d, *J* = 8.1 Hz, 1H, *ortho*-4-Ph), 7.24 (d, *J* = 8.4 Hz, 2H, *para*-3,5-Ph), 7.32 (s, *ortho*-2-Ph), 7.34 (m, 20H, Ph (Bd)), 7.50 (t, *J* = 7.6 Hz, 1H, *ortho*-5-Ph), 7.82 (d, *J* = 8.3 Hz, 2H, *para*-2,6-Ph), 7.93 (d, *J* = 7.6 Hz, 1H, *ortho*-6-Ph). ^13^C{^1^H} NMR (DMSO, *δ*, ppm): 126.67, 127.53, 128.03, 128.52, 128.78, 129.60, 130.18, 130.37, 130.49, 131.42, 133.09, 135.23, 136.82 (all s, Ar + Ph), 146.48, 148.48 (two s, SC=N), 157.40, 157.48 (two s, PhC=N), 167.27, 166.57 (two s, COOH). UV-Vis (CH_3_OH) *λ*_max_, nm (*ε* × 10^−3^, L mol^−1^ cm^−1^) 245(31), 292(15), 365(4.5), 429(4), 454(9), 483(11), 506(11).

#### 2.2.3 FeBd_2_(*para*,*meta*-(HOOCC_6_H_4_S)_2_Gm)(BF)_2_ (6)

Yield: 55%. Calcd for C_44_H_30_N_6_B_2_F_2_FeO_10_S_2_·0.5H_2_O: C, 53.31; H, 3.15; N, 8.48. Found (%): C, 53.30; H, 3.32; N, 8.30. ESI-LC/MS (acetonitrile): *m*/*z*: 965 [M without hydroxygroup]^+^, 982 [M]^+^˙. ^1^H NMR (DMSO, *δ*, ppm): 7.21 (d, *J* = 8.4 Hz, 2H, 2,6-Ph), 7.39 (d, *J* = 3.9 Hz, 20H, Ph (Bd)), 7.47 (t + s, *J* = 7.8 Hz, 1 + 1H, *meta*-2,5-Ph), 7.63 (d, *J* = 7.8 Hz, 1H, *meta*-6-Ph), 7.76 (d, *J* = 7.9 Hz, 1H, *meta*-4-Ph), 7.80 (d, *J* = 8.4 Hz, 2H, *para*-3,5-Ph). ^13^C{^1^H} NMR (DMSO, *δ*, ppm): 128.07, 128.49, 128.57, 128.82, 129.07, 129.22, 129.39, 130.02, 130.51, 130.82, 130.90, 130.97, 132.54, 132.90, 133.65, 137.15 (all s, Ar + Ph), 146.72, 147.90 (two s, SC=N), 158.11, 158.24 (two s, PhC=N), 166.81, 166.96 (two s, COOH). UV-Vis (CH_3_OH) *λ*_max_, nm (*ε* × 10^−3^, L mol^−1^ cm^−1^): 235(30), 269(14), 292(6), 390(3), 450(6), 471(12), 503(10).

### 2.3. Fluorescent spectroscopy

0.05 M Tris–HCl aqueous buffer with pH 7.9 was used for the preparation of BSA, HSA, BLG, LYZ and trypsin solutions with *c*_protein_ = 3 μM; an aliquot of the freshly prepared 2 mM DMSO solution of the clathrochelate under study was added to this protein buffer solution. Since an amount of the above DMSO solution was rather small (up to 0.8% of a total volume), the protein concentration almost persisted, while the concentration of the clathrochelate has been changed from 1 to 15 μM.

To obtain an idea of the values of equilibrium constants of clathrochelates binding to proteins, binding stoichiometry and binding affinity were estimated for HSA and two of the clathrochelate complexes (1 and 4). For this, titration of HSA by the compounds 1 and 4 was performed three times, and the average values were used. Estimation was performed under the assumption that each protein globule has *n* sites for clathrochelate binding with the equal values of the binding constant *K*. To obtain the values of *K* and *n*, the experimentally obtained curve of protein fluorescence quenching is presented as the dependence of (1 − *F*/*F*_0_) (where *F*_0_ and *F* are protein fluorescence intensities in the absence and in the presence of clathrochelate) on clathrochelate concentration (*C*_L_), and further fitted with the dependence (1) (obtained as described in ESI†):1
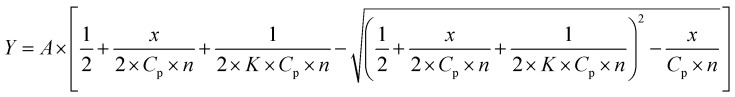
where *x* = *C*_L_, *Y* = (1 − *F*/*F*_0_), *A* = (1 − *F*_min_/*F*_0_), *F*_min_ is the protein fluorescence intensity upon all the binding sites occupied with clathrochelate molecules, *C*_P_ is the concentration of protein globules. As a result of this fitting, the values of *K*, *n* and *A* are obtained as fitting parameters; besides, the values of *K* and *A* were also obtained upon the fixed values on *n* = 1.

### 2.4. Corrections on an inner filter effect and reabsorption

Addition of the clathrochelates substantially enhances the optical densities of the solutions at the wavelengths of the protein fluorescence excitation and emission thus decreasing the protein fluorescence intensity due to the “inner filter effect” (IFE) and the reabsorption process. To avoid the errors caused by IFE and reabsorption, the intensities of the protein fluorescence spectra in the presence of these cage complexes were corrected using eqn (2).^21,22^

2 *F*_cor_ = *F*_obs_ × 10^(*D*_ex_+*D*_em_)/2^

where *F*_cor_ and *F*_obs_ are the corrected and observed fluorescence intensities, respectively; *D*_ex_ and *D*_em_ are the clathrochelate's optical densities at the wavelengths of protein excitation and emission, respectively.

### 2.5. Circular dichroism spectroscopy

The CD spectra were recorded on a Jasco J-1500 spectropolarimeter at room temperature in 300–600 nm range; three scans were averaged for each of the ICD spectra. The data are expressed as ellipticity (mdeg), obtained in mdeg directly from the instrument. Tris–HCl aqueous buffer (pH 7.9) was used for the preparation of the stock solutions of proteins, as well as of the working solutions with protein-to-clathrochelate 2 : 1 molar ratio (*c*_protein_ = 4 × 10^−5^ mol l^−1^, *c*_clt_ = 2 × 10^−5^ mol l^−1^). It should be noted that the fluorescence quenching was rather low at this molar ratio. However, due to the peculiarities of the CD and fluorescent experiments, the absolute concentrations used in CD studies (4 × 10^−5^ mol l^−1^ for proteins) were substantially higher, as compared to those used in the fluorescence studies (*c*_protein_ = 3 × 10^−6^ mol l^−1^). Thus, according to the mass action law, a higher percentage of the clathrochelate molecules is bound to proteins at the molar ratio 2 : 1 under the conditions of the CD experiment, as compared to those of the fluorescent experiments. One more difference is that in the fluorescent experiment, high percentage of proteins bound to clathrochelates is important (*i.e.* excess of clathrochelate molecules is required), while in the CD experiment high percentage of bound clathrochelates is essential (*i.e.* excess of protein molecules is required). Due to these two reasons, the induced CD output was clearly observed at the used molar ratio.

At the same time, in order to study the possibility of inducing CD response upon binding to the (possible) protein sites with lower affinity, we have also studied CD spectra of clathrochelates in the presence of BSA, HSA, BLG and LYZ with protein-to-clathrochelate 1 : 2 molar ratio (*c*_protein_ = 10^−5^ mol l^−1^, *c*_clt_ = 2 × 10^−5^ mol l^−1^).

### 2.6. Study of the BLG–clathrochelate 3 assembly by MALDI-TOF mass-spectrometry

The corresponding spectra were obtained on a Bruker Daltonics MALDI-TOF mass spectrometer. The sample was prepared by mixing 50 μl of BLG solution (2 mg ml^−1^ in 0.1 M aqueous ammonium citrate, pH adjusted to 7.5 with NH_4_OH_aq_) with 50 μl of a methanol solution of this complex (1.7 mg ml^−1^), thus giving a BLG-to-clathrochelate 1 : 15 molar ratio. This probe was applied onto the nickel plate.

### 2.7. Quantum-chemical simulation of CD spectra

To decipher ICD spectra, we used the TD-DFT computational method, as the most widely used and the most cost-efficient, for the theoretical prediction of UV-VIS and ICD spectra. An assignment of the molecular structures and the predictions of their UV-VIS spectra has been earlier successfully performed^23^ for the clathrochelate complexes using this method.

The geometries of both the *Δ* and *Λ* conformations of a clathrochelate molecule were optimized by wB97X functional with RIJCOSX approximation and the calculation of the first 50 excited states were performed with PBE0 functional (def2-TZVP basis set) with ORCA program suit (version 4.1.2). The calculated peaks were broadened in ChemCraft program, thus giving the spectra which were similar to those experimentally measured.

### 2.8. Molecular docking calculations of a binding of the clathrochelate complexes 1 and 2 to BLG

The docking simulations were performed using AutoDock Vina program (1.1.2, The Scripps Research Institute, La Jolla, CA, USA) along with MGLTools (1.5.6, The Scripps Research InstituteManufacturer, La Jolla, CA, USA).^24,25^

The molecular structure of clathrochelate 1 was obtained using a manual treatment of the known^12^ single crystal XRD structure of clathrochelate 2, and both of them were optimized by wB97X-D3 method with def2-TZVP basis set^26–29^ using ORCA 4.0.1 program suit;^30,31^ their non-polar hydrogen atoms were merged and the rotatable bonds were defined. The corresponding deprotonated macrobicyclic structures were obtained using an elimination of hydrogen atoms of their COOH groups and assigning the Gasteiger charges.

Crystal structure of BLG (PDB ID: 2BLG) was obtained from Protein Data Bank,^32^ the solvent and ligand molecules were removed. Then, when all hydrogen atoms were added, the Gasteiger charges were computed and the C–H hydrogen atoms were merged. The optimal calculation size for evaluation of the above clathrochelate molecule was calculated as described^33^ as the cube with the length of the edge of 23 Å.

The coordinates of the main binding site of BLG were defined as a centre of the cavity (the crossroad of the residues ILE72, ASN90, LEU39, LEU58): centre = −4.6, centre_*y* = 8.7, centre_*z* = 19.5 in the coordinate system of the original pdb file from RSCB protein data bank, the sizes of grid box were set as follows: size *x* = 25, size *y* = 40, size *z* = 25.

The value of an exhaustiveness was set to 100. For Vina docking, the default parameters were used. All the calculations were repeated at least twice. The best-scoring pose as judged by the Vina docking score was chosen and visually analyzed using MGLTools.

## 3. Results and discussion

### 3.1. Synthesis

**Scheme 2:**
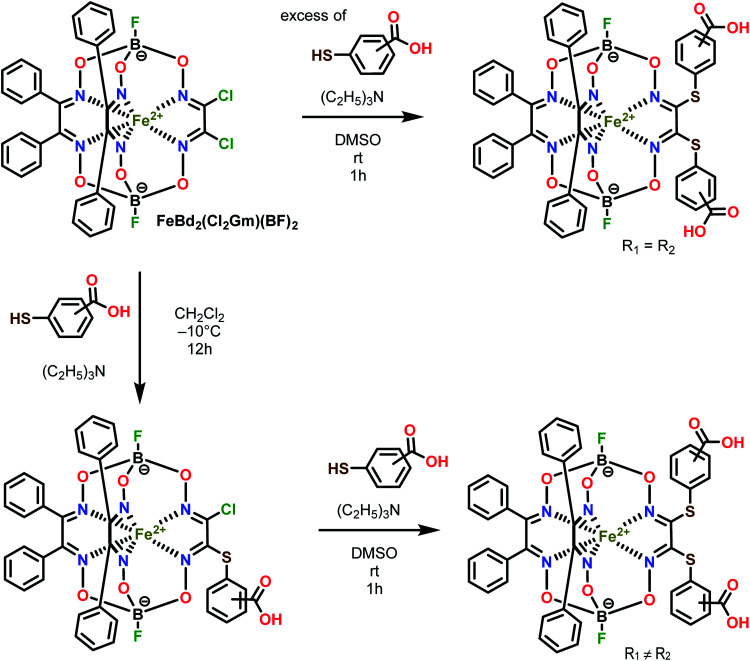
Synthesis of the dicarboxyphenylsulfide iron(ii) clathrochelates with equivalent (on top) and non-equivalent (on bottom) ribbed substituents. R_1_ ≠ R_2_.

Three homodifunctionalized cage complexes FeBd_2_((X-HOOCC_6_H_4_S)_2_Gm)(BF)_2_ (1–3, where Bd^2−^ is α-benzildioxime dianion, Gm is glyoxime residue, BF is the fluoroboron capping group) with equivalent *ortho*-, *meta*- or *para*-substituted arylsulfide ribbed groups were easily obtained in dichloromethane as a solvent at r.t. by Scheme 2 using nucleophilic substitution of their dichloroclathrochelate precursor FeBd_2_(Cl_2_Gm)(BF)_2_ with 1.5-fold excess of the corresponding mercaptobenzoic acid. So, the clathrochelate precursor-to-nucleophile ratio was equal to 1 : 3, thus allowing to perform a complete functionalization of the above macrobicyclic precursor with two ribbed chlorine atoms. In the case of three heterodifunctionalized cage complexes FeBd_2_(X,Y-(HOOCC_6_H_4_S)_2_Gm)(BF)_2_ (4–6, where X, Y- are *ortho*, *para*-; *meta*, *para*-; or *ortho*, *meta*-positions of their terminal carboxyl groups), a subsequent addition of one equivalent of the first nucleophile (*i.e.* the corresponding carboxyphenylthiolate anion) to the dichloromethane solution of FeBd_2_(Cl_2_Gm)(BF)_2_ and one equivalent of the second nucleophile, its constitutional isomer, in DMSO as a solvent was used. The mixtures of the arylsulfide clathrochelate products of the above substitution reactions were separated by column chromatography.

The complexes obtained were characterized using elemental analysis, ESI-LC/MS mass spectrometry, IR, UV-Vis, ^1^H and ^13^C{^1^H} NMR spectra. The most intensive peaks in the positive range of their MALDI-TOF mass spectra belong to the corresponding molecular ions.

The solution ^1^H and ^13^C{^1^H} NMR spectra (ESI, Fig. S1–S3†) of monoribbed-functionalized clathrochelates 4–6 contain the signals of both their ribbed phenyl and carboxyl-terminated arylsulfide substituents in α-dioximate chelate fragments. The number and position of the signals in these spectra, as well as the ratio of their integral intensities in the ^1^H NMR spectra, confirmed the composition and the symmetry of these macrobicyclic molecules. In their ^13^C{^1^H} NMR spectra, the signals of two and four types of azomethine carbon atoms were observed for the cage complexes with equivalent (1–3) and non-equivalent (4–6) ribbed substituents, respectively. This indicates an absence of the symmetry plane in the molecules 4–6 that passes through the middles of the chelate C–C bonds in the above chelate fragments and the encapsulated iron(ii) ion as well. A doubling of the signals of carbon atoms in their α-benzildioximate ribbed moieties was also observed in these spectra.

### 3.2. Fluorescent spectra

**Fig. 2:**
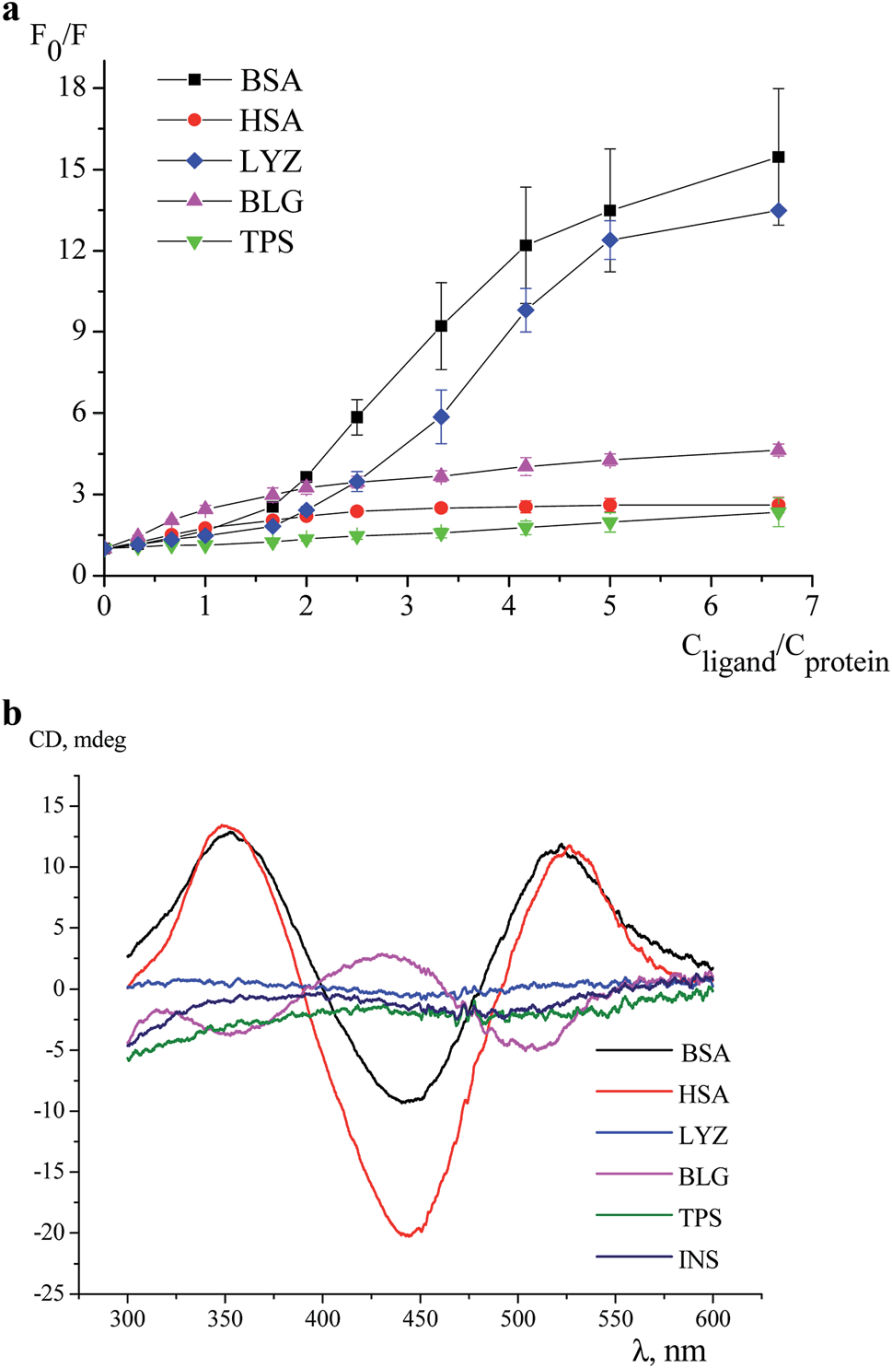
Stern–Volmer plots of the fluorescence quenching of BSA (excitation at 280 nm, emission at 344 nm), HSA (emission at 353 nm), LYZ (emission at 344 nm), BLG (emission at 336 nm) and of TPS (emission at 343 nm) by the clathrochelate 6 with a standard deviation as the error bars: *c*_protein_ = 3 μM, 0.05 M Tris–HCl buffer with pH 7.9 used as a solvent at 25 °C (A) and ICD spectra of its assembly with the proteins BSA, HSA, LYZ, BLG, TPS and INS (*c*_protein_ = 40 μM; *c*_clathrochelate_ = 20 μM), in 0.05 M Tris–HCl buffer with pH 7.9 at 25 °C (B).

An effect of iron(ii) clathrochelates 1–6 on the intrinsic fluorescence of a series of proteins BSA, HSA, LYZ, BLG and TPS (Fig. 1) was studied to characterize the corresponding clathrochelate–protein assemblies. For this, the Stern–Volmer plots of protein fluorescence intensities *versus* the clathrochelate-to-protein molar ratios were obtained (Fig. 2 and S4–S8, see ESI†); the values of a quenching of the proteins' intrinsic fluorescence are summarized in Table 1. A protein fluorescence quenching upon an addition of these clathrochelates suggests the protein-to-clathrochelate binding that caused the changes in a closest environment of the proteins' Trp residues.^15,34^

**Table 1:** Values of a quenching of the protein intrinsic fluorescence (*F*_0_/*F*) at a protein-to-clathrochelate molar ratio 5 : 1

Compound	BSA	HSA	LYZ	BLG	Trypsin
1	9.9	2.5	10.4	3.4	1.2
2	16.8	2.2	12.7	3.8	1.2
3	9.3	1.6	10.1	3.6	1.3
4	11.8	3.0	10.3	3.5	1.4
5	9.0	2.1	9.8	3.2	1.2
6	12.5	2.2	11.6	3.4	1.2

In this work, we studied interactions of the designed carboxyl-terminated iron(ii) clathrochelates with a series of globular proteins, the molecules of which contain the Trp residues and have the binding sites of different functionality (those for transport of the small molecules or the enzymatic cavities). Since insulin molecule does not contain the fluorescently sensitive Trp residues,^35^ this protein was not used in the fluorescence quenching studies; on the other hand, the above clathrochelates were tested to give an ICD output in the presence of this hormone.

The BSA and HSA molecules contain two main drug binding sites (*i.e.* Sudlow I and II), possessing similar structures with positively charged Lys and Arg amino acid residues. However, these protein macromolecules have different number of Trp residues (Fig. 1) and, therefore, used as hosts, they demonstrated different fluorescent responses upon clathrochelates binding. Indeed, BSA molecule contains two Trp residues: one, Trp 134, located on a surface of the protein globule and “opened” to an effect of the medium factors, and second, Trp 213, hidden inside of the globule and, therefore, less available. HSA contains only one, hidden Trp 214 residue.^36^

In agreement with these structural distinctions, we observed that the clathrochelates quench the fluorescence of BSA and HSA with substantially different efficiencies (Table 1). In particular, quenching of BSA emission (9–17-fold) is substantially more pronounced than that of HSA (1.6–3-fold). Besides, different shifts of the emission maxima upon protein–clathrochelate interactions, *i.e.* up to 38 nm to the blue range for BSA (ESI, Fig. S11a†) and less than 9 nm to the red for HSA (ESI, Fig. S11b†) were observed. In general, the efficiency of the quenching of protein fluorescence was only slightly affected by the constitutional isomerism of the clathrochelate. The profiles of quenching curves in the case of albumins are similar, except of those for the assemblies of 2 with BSA and 3 with HSA. To estimate the affinity of the studied clathrochelates to proteins, binding stoichiometry (*n*) and binding constant (*K*) were estimated for the interaction of compounds 1 (containing equivalent substituents) and 4 (containing non-equivalent substituents) with HSA. For the estimation, we used the model implying the presence of *n* binding sites in protein globule with equal values of *K* and equal impact on the protein fluorescence (see subsection 2.3 above and Section 5 in ESI†). The estimation (based on the rather good fitting) resulted in close values of *K* equal to (1.7 ± 0.2) × 10^6^ M^−1^ and (1.5 ± 0.3) × 10^6^ M^−1^, respectively for the compounds 1 and 4 (Fig. S9a and S10a, see ESI†); the number of clathrochelate molecules bound per HSA globule (*n*) was calculated as 0.52 ± 0.05 and 0.43 ± 0.08, respectively for 1 and 4. One of the possible ways to explain such obtained values of *n* (which do not have sense in the frames of the used model and are thus only apparent ones) could be the availability of several binding sites in HSA molecule with different values of the binding constant and different degree of protein fluorescence quenching when occupied; this means that the used model of several binding sites with equal binding constants and equal degree of fluorescence quenching is not appropriate for the binding of 1 and 4 to HSA. We have also estimated the values of *K* upon the fixed values on *n* = 1 (Fig. S9b and S10b, see ESI†), the values of *K* equal to (8 ± 5) × 10^6^ M^−1^ and (8 ± 6) × 10^6^ M^−1^ respectively were obtained for the compounds 1 and 4 (but the fitting poorly correspond to experimental data). Hence, for the clathrochelates containing equivalent and non-equivalent substituents, the values of the binding constant are close.

LYZ, a protein with enzymatic activity, contains six Trp residues (Fig. 1) and two of them, Trp 62 and Trp 108, are responsible for approximately 80% of its intrinsic fluorescence.^37^ Therefore, if a binding of the small guest molecules to LYZ quenches its fluorescence, the corresponding hosting sites are near these two Trp residues.^37,38^ In LYZ active centre, two of the six Trp residues (Trp 62 and Trp 63) are located. Besides, the positively charged Arg 61 residue is located nearby of them and it is able to interact with terminal carboxyl groups of a clathrochelate molecule. At the same time, another residue, Arg 112, that is able to interact with these carboxyl groups, is located near other tryptophan residues, Trp 108 and Trp 111. So, we suggest that binding of a clathrochelate molecule occurs into (or nearby) these two LYZ motifs, leading to the quenching of the protein fluorescence.

Binding of iron(ii) clathrochelates 1–6 to LYZ was evidenced by a substantial (10–13-fold) decrease of protein emission upon their assembly, whereas the corresponding emission maxima persisted; the shapes of the corresponding quenching curves are similar for all cage compounds (Fig. S6†). Strong decrease in LYZ fluorescence upon binding of clathrochelates evidenced that the metal complexes affect the most fluorescent Trp residues, thus binding to both above motifs (*i.e.* near Trp 62 and/or Trp 108 residues) may be suggested.

The protein of a lipocalin family, BLG (Fig. 1), is known to be able to bind small molecules and transport them to biological systems.^39^ Its macromolecule contains two Trp residues: Trp 19 located inside the hydrophobic pocket, and Trp 61 located on its surface near this pocket.^39^ Two positively charged Lys residues (Lys 60 and Lys 69) are located near the entrance to a hydrophobic calix, known as the main guest's binding site of BLG.^15^ Upon addition of iron(ii) clathrochelates 1–6, unsubstantial quenching of the protein fluorescence with a close range of *F*_0_/*F* values (3.2–3.8-fold), and a slight (up to 4 nm) red shift of the protein emission maxima were observed. As in the case of LYZ, the quenching patterns of BLG fluorescence were only slightly affected by a constitutional isomerism of the above iron(ii) clathrochelates with terminal carboxyl groups.

TPS contains four Trp residues (Fig. 1),^40,41^ which are located outside of its catalytic centre that is negatively charged by Asp 189 residue.^42^ Addition of iron(ii) clathrochelates 1–6 to TPS caused an almost negligible (1.2–1.4-fold) quenching of its protein fluorescence without a shift of the corresponding emission maxima (Fig. S8†). This suggests either weak TPS–clathrochelate interactions, or a remoteness of the binding site of clathrochelate reporters from the Trp residues of this protein.

So, at the above first stage of our experiments, we studied the interactions of the designed clathrochelates with globular proteins, the molecules of which contain the fluorescent Trp residues.^35^ The fluorescence quenching studies evidenced the binding of the disubstituted carboxyphenylsulfide iron(ii) clathrochelates to the proteins, specifically serum albumins, LYZ and BLG. These proteins contain nearby their binding sites the positively charged Arg and Lys residues, which are able to form electrostatic (polar) interactions with the above cage molecules. Therefore, the corresponding Trp residues, located in close proximity to the bindings sites, are affected by the assembly with the clathrochelates, thus giving the corresponding spectral output. Constitutional isomerism of the clathrochelate substantially affected the degree of quenching of the intrinsic protein emission in the case of albumins, being, however, substantially less pronounced for LYZ and BLG.

### 3.3. ICD spectra

For the first time, an induction of the CD signal upon a clathrochelate-to-protein interaction has been observed for the carboxyphenylsulfide iron(ii) clathrochelates with two equivalent ribbed substituents in their assemblies with BSA.^16^ At this stage, we tested an ICD output caused by a binding of all six constitutional isomers of the dicarboxylphenylsulfide iron(ii) clathrochelate to a series of globular proteins.

**Fig. 3:**
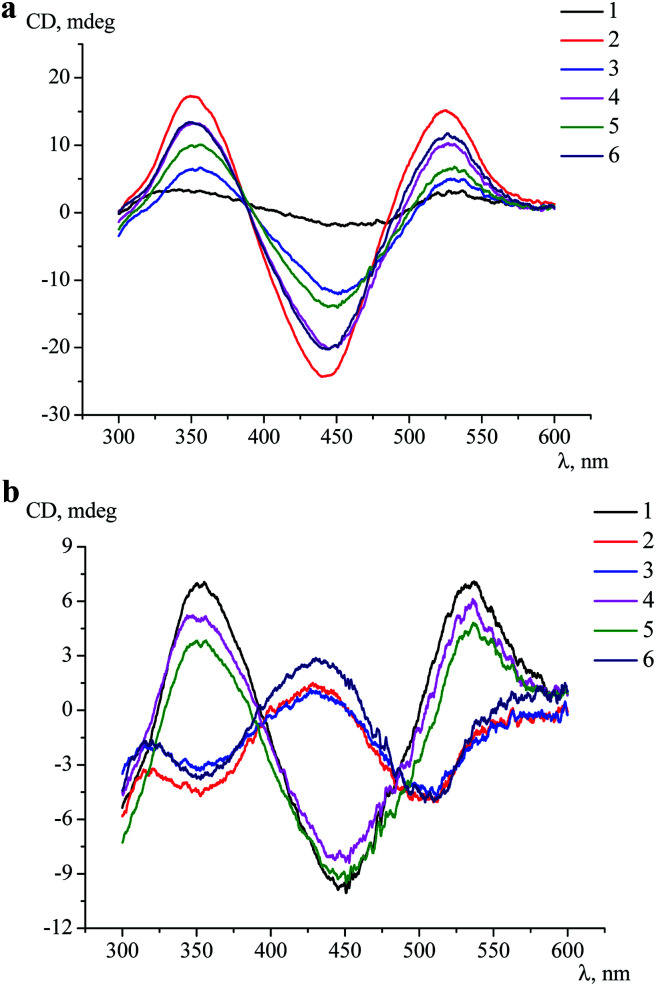
CD spectra of the assemblies of proteins HSA (a) and BLG (b) with the clathrochelates 1–6 measured at *c*_protein_ = 4 × 10^−5^ mol l^−1^ and *c*_clt_ = 2 × 10^−5^ mol l^−1^ in 0.05 M Tris–HCl buffer with pH 7.9 at 25 °C.

Clathrochelates 1–6 are inherently CD-silent, whereas their binding to serum albumins caused an appearance of strong ICD bands possessing similar shape with two positive (at approximately 350 and 520 nm) and one negative (at approximately 450 nm) bands of different intensities (Fig. 3a). These CD signals were characterized as a sum of the moduli of maximum-to-minimum band intensities (ΔICD) in the 300–600 nm range (Table 2).

**Table 2:** ΔICD values (mdeg) for the iron(ii) clathrochelates under study induced in the presence of globular proteins (*c*_protein_ = 4 × 10^−5^ mol l^−1^, *c*_clt_ = 2 × 10^−5^ mol l^−1^, 0.05 M Tris–HCl buffer with pH 7.9 at 25 °C)

Compound	Initial complex	Initial complex +					
		BSA	HSA	LYZ	BLG	Trypsin	Insulin
1	0.9	28.9	5.1	1.6	16.6	1.1	1.8
2	1.0	40.4	42.5	1.6	6.2^a^	1.9	1.6
3	0.9	19.3	18.8	0.5	5.3^a^	1.7	2.1
4	0.9	37.5	33.2	2.9	13.3	1.5	1.6
5	1.1	12.1	23.6	3.2	12.6	1.8	2.0
6	1.0	21.8	33.2	1.3	7.7^a^	0.7	1.6

a This band has an opposite sign (Fig. 3b).

As it can be seen from Table 2, the constitutional isomerism of the clathrochelate substantially affected the intensity of the corresponding ICD outputs, which were in a wider range (5–42.5 mdeg) in the case of HSA, as compared with BSA (12–40.4 mdeg). The most intensive spectral output upon binding to albumins was observed for di-*meta*-substituted cage complex 2 (40.4 and 42.5 mdeg for BSA and for HSA, respectively); the intensities of CD bands for clathrochelates 3 and 4 in the presence of both these albumins were similar. At the same time, di-*ortho*-functionalized analogue 1 strongly discriminated between BSA and HSA, inducing more than 5-fold more intensive CD signal upon binding to BSA than to HSA. On the other hand, the ICD signal for complex 5 is about 2-fold more intensive when assembled with HSA, as compared to BSA (Table 2).

Despite the substantial differences observed in the intensities of the corresponding ICD bands, the shape of the spectra was almost the same, thus suggesting similar character of the TP–TAP (trigonal prismatical–trigonal antiprismatical) distortions of the rigid quasiaromatic cage frameworks of clathrochelate molecules 1–6 upon their assembly with albumins.

In order to study a possibility to induce a CD output upon the clathrochelate binding to protein sites of a lower affinity, we measured the CD spectra (Fig. S12 and S13, see ESI†) of macrobicyclic complexes 1–6 in the presence of albumins, but with an excess of the clathrochelate, *i.e.* at protein-to-clathrochelate molar ratio 1 : 2, thus using the same concentration of the clathrochelate and four times lower concentration of the protein (as compared to the previous set of experiments). For the assemblies of 4, 5 and 6 with HSA, as well as 2, 4 and 5 with BSA, passing from an excess of protein to an excess of clathrochelate did not affect the shape of the spectrum, leading only to a decrease of the signal amplitude ΔICD (Fig. S12 and S13, see ESI†). Thus, for these clathrochelate–protein pairs, the decrease in protein concentration led to the decrease in the number of clathrochelate molecules bound to the “CD-inducing” sites; the other clathrochelate molecules could either remain in solution, or bind to the sites of a lower affinity, which do not induce the CD output. Here we should emphasize the *S*-like shape of albumins fluorescence quenching curves that is clearly observed for the assemblies of all the cage guests with BSA, and 5 with HSA, being much less pronounced for other clathrochelate–HSA pairs (Fig. S4 and S5, see ESI†). One of the possible explanations of the above mentioned *S*-like shape (together with CD results) could be the existence of two types of BSA/HSA protein sites (with higher and lower binding affinity) that could be occupied with clathrochelate molecules. In the frame of this explanation, the binding of the clathrochelate to the site with higher affinity (that occurs at low clathrochelate-to-protein concentration ratios) is thus suggested to induce strong CD response, but only a weak quenching of Trp fluorescence. At the same time, clathrochelate binding to the lower affinity sites of the BSA/HSA proteins (that occurs at higher clathrochelate-to-protein concentration ratios) are supposed not to induce the CD response, but cause stronger protein fluorescence quenching. In parallel, upon the binding of 1, 2 and 3 and HSA, as well as 1, 3 and 6 with BSA, a decrease in protein concentration was accompanied by a small (2–6 nm) shift of the band with the maximum at 450 nm (Fig. S12 and S13, see ESI†). So, in these cases, the binding of the clathrochelates to the lower affinity sites, leading to both the fluorescence quenching and low induction of the CD output, is suggested.

**Fig. 4:**
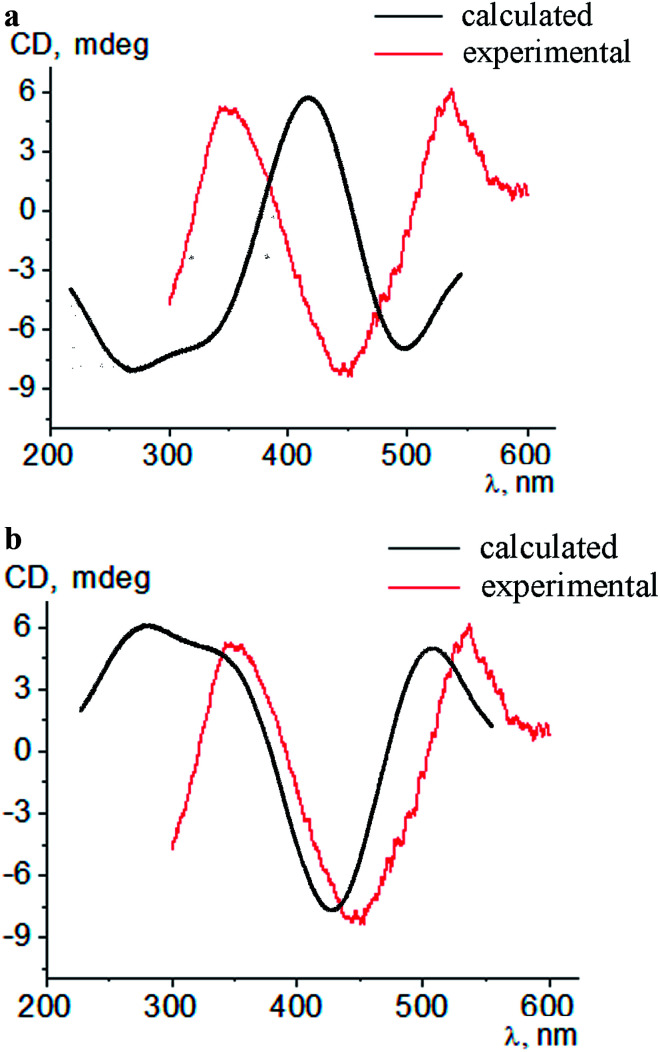
Simulated and experimental CD spectra of the conformations *Δ* (a) and *Λ* (b) of a macrobicyclic framework of the *meta*,*ortho*-substituted dicarboxyphenylsulfide iron(ii) clathrochelate 4 in the presence of BLG.

Intensive (5.3–16.6 mdeg) ICD responses on the clathrochelates binding were also observed for BLG (Fig. 3b). Cage iron(ii) complexes which contain at least one terminal *ortho*-carboxyl substituent (*i.e.* complexes 1, 4 and 5) upon their binding to BLG gave the ICD spectra with the shape similar to this of their assemblies with albumins. On the other hand, the binding of the macrobicyclic complexes 2, 3 and 6, with *meta*- and *para*-carboxyl group(s), induced the CD bands of an opposite sign (Fig. 3), that can be explained by a fixation of two opposite (*Λ* or *Δ*) TP–TAP-distorted conformations of the clathrochelate framework.^16^ The simulated spectra of these two conformers of clathrochelate 4 are shown in Fig. 4. A comparison of the spectra simulated for 4 with those experimentally obtained for 1–6 suggests, in most cases, a stabilization of the *Λ* conformation of the macrobicyclic framework in the protein–clathrochelate assembly (Fig. 3 and 1b). Interactions of the cage complexes with BLG caused the stabilization of *Δ*-conformation for 2, 3 and 6, and *Λ*-conformation for 1, 4 and 5. It should be noted that passing from 2 : 1 to 1 : 2 BLG-to-clathrochelate ratio did not affect the shape of ICD spectra of these clathrochelates 1–6 in the assemblies with BLG.

With LYZ as a host, an induction of the clathrochelate chirality was rather weak and the intensities of ICD bands were in the range of 0.5–3.2 mdeg; the most intensive signal was observed for clathrochelate 5. Low intensities of ICD bands and strong quenching of LYZ fluorescence caused by the clathrochelates (see above) suggest either a flexibility of their cage frameworks in the protein–clathrochelate assemblies, or a low “selectivity” of the LYZ binding site to a given conformation (*Λ* or *Δ*).

The intensities of the clathrochelate ICD bands in the presence of hormone insulin, as well as trypsin, which contain negatively charged catalytic sites,^43^ were negligible and similar to those for the initial cage iron(ii) compounds.

So, the interactions of the isomeric dicarboxylphenylsulfide iron(ii) clathrochelates with two equivalent or non-equivalent ribbed substituents with proteins caused an induction of intensive CD outputs in the case of serum albumins and BLG, which contain well-formed hydrophobic binding pockets and nearby positively charged amino acid residues. The constitutional isomerism of ribbed substituents of the macrobicyclic reporter molecule affected the intensities of the corresponding clathrochelate ICD outputs and is responsible for the “selection” of the predominant optically active left- or right-handed-distorted (*Λ* or *Δ*) conformations of the cage framework that is stabilized by its interactions with the binding site of the protein.

### 3.4. MALDI-TOF characterization of the BLG–clathrochelate 3 assembly

**Fig. 5:**
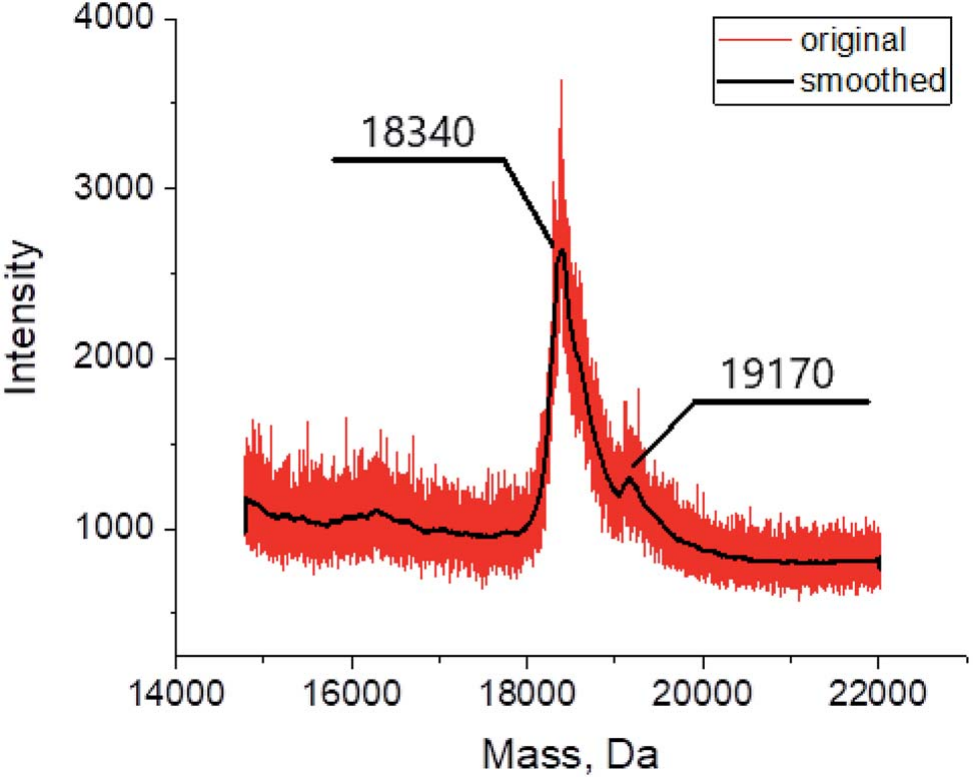
MALDI-TOF spectrum of the BLG–clathrochelate 3 assembly.

The MALDI mass spectrometry was used to confirm the formation of the assembly between the BLG and cage complex 3, chosen here as an example. The mass spectrum of the corresponding probe (Fig. 5) contains in the mass range 18 000–20 000 Da an intensive peak with *m*/*z* of 18 340 assigned to the initial protein macromolecule, as well as that of a lower intensity with *m*/*z* of 19 170 assigned to the BLG–clathrochelate 3 1 : 1 assembly. Such a low intensity of the latter peak was observed even for the samples with a high, 15-fold, molar excess of 3; this result may be explained by a low stability of the assembly upon its ionization.

### 3.5. Molecular docking of a clathrochelate binding to BLG

Because the binding of clathrochelates 1 and 2 to BLG induced the CD signals of the opposite signs, we have chosen these complexes for a molecular docking simulation of the interactions of their different conformations *Λ* and *Δ* with the protein.

BLG with its molecular mass of 18.3 kDa contains 162 amino acid residues and its main binding site is postulated to be at its calyx entrance. An accessibility of the calyx for a guest molecule is known^44^ to be a pH dependent: at pH lower than 6.5, the EF loop, a motif of this macromolecule containing the residues from I 84 to N 90, remains closed and it opens up at pH >7.^44^

The results of the performed molecular docking suggest that each of the clathrochelate molecules is able to fit the entrance of the BLG cavity, in which it forms the bonds with amino acid residues of the protein (mainly through the non-polar dispersion interactions). To check an ability of the polar interactions between the amino acid residues and the clathrochelate molecule, we also performed its docking procedure with the residues Leu 46, Leu 54, Ile 56, Ile 71, Ile 84, Phe 105, Met 107, Lys 60, and Lys 69 set as flexible. The simulations showed no formation of the coulombic (polar) interactions, while they suggest different values of the binding energy for the optically active *Λ* and *Δ* conformations of the clathrochelate 1 and 2 molecules (Table 3). Therefore, the dispersion forces could make impact into the chiral induction observed (*i.e.* the stabilization of a given TP–TAP-distorted framework). On the other hand, the terminal carboxyl groups of the clathrochelate molecule and lysine residues are visually in close proximity, thus making it possible that the polar interactions can play the role of an anchor for this cage molecule in its assembly with the protein.

**Table 3:** Calculated energies of *Δ* and *Λ* conformations of the clathrochelate molecules 1 and 2

1(*Δ*)	1(*Λ*)	2(*Δ*)	2(*Λ*)
−9.5	−9.2	−9.8	−8.8
1(*Δ*) deprotonated	1(*Λ*) deprotonated	2(*Δ*) deprotonated	2(*Λ*) deprotonated
−9.7	−9.4	−10.4	−8.9

**Fig. 6:**
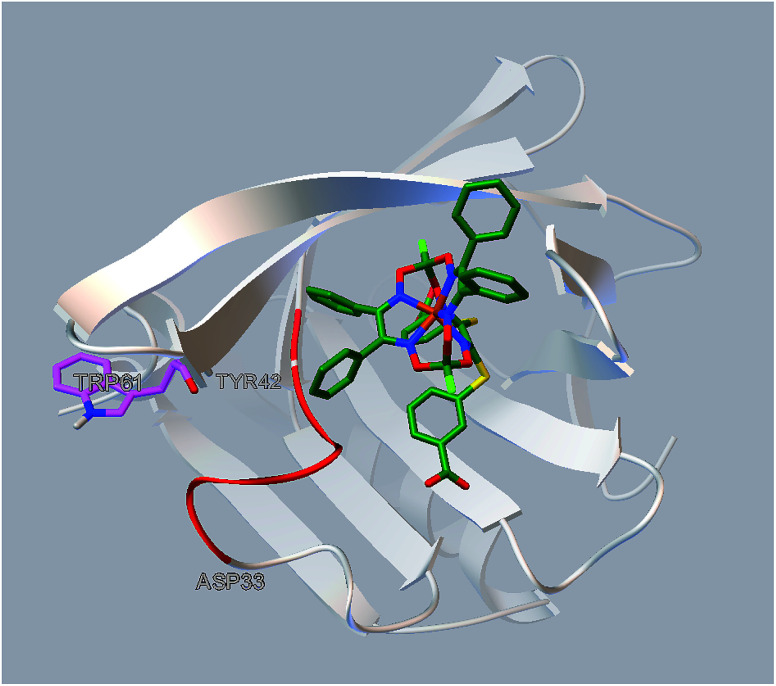
Localization of the clathrochelate guest 2 in a proximity of the Trp 61 residue of BLG (as it deduced from the molecular docking calculations).

The simulated site of the clathrochelate binding is also in a close proximity to the fluorescent Trp 61 residue (Fig. 6). This site is connected by a flexible sequence Ala 34–Tyr 42 and its movement upon a binding of the corresponding clathrochelate can directly lead to change of the Trp 61 environment and, therefore, cause the decrease in a protein fluorescence; this quenching is described above (Section 3.2).

## 4. Conclusions

With the aim to design the functionalized iron(ii) clathrochelates as ICD protein-sensing reporters, we developed the synthetic approach for the modification of one chelate α-dioximate fragment of the macrobicyclic tris-dioximate framework with two non-equivalent ribbed substituents. This allowed to obtain a series of constitutional isomers of iron(ii) clathrochelate with two non-equivalent carboxyphenylsulfide substituents. The ability of symmetric and non-symmetric di-carboxyphenylsulfide clathrochelate isomers to form the assemblies with a series of globular proteins – serum albumins, LYZ and BLG was confirmed by protein fluorescence quenching and CD experiments. Molecules of these proteins contain the hydrophobic cavities and nearby positively charged Arg or Lys amino acid residues, which are suggested to promote the formation of the protein–clathrochelate assemblies through electrostatic (polar) interactions. The binding constants (*K*) for clathrochelate–HSA assemblies estimated by protein fluorescence quenching method have rather high values – about 10^6^ M^−1^. The highest CD output of clathrochelates was observed in the presence of albumins and BLG. In the case of albumins, the constitutional isomerism of clathrochelate affected only intensities of ICD spectra, while the shape of the spectra persisted. This behaviour suggests the stabilization of the clathrochelate framework in only one, *Λ*-conformation. In the case of BLG the constitutional isomerism determines the shape of ICD spectra: *ortho*-carboxyl-terminated clathrochelate isomers showed the opposite signs of the ICD bands, as compared to those of *meta*- and *para*-carboxyl-terminated ones. This inversion of the band sign suggests the stabilization of opposite configurations (*Λ* or *Δ*) of the clathrochelate framework in the assembly with BLG. So, dependently from constitutional isomerism of the terminal substituents, the protein could select between the opposite configurations of clathrochelate framework. MALDI TOF results show the formation of BLG–clathrochelate complex with ratio 1 : 1. The docking simulations point on location of the clathrochelate molecule in the main BLG binding site (calyx) in its open conformation. The above results suggest the prospects of using the clathrochelate framework as the molecular scaffold for the design of protein-sensitive ICD reporters, and the efficiency of proposed approach of the variation of ribbed substituents to achieve the specificity of ICD response to the target protein.

## Conflicts of interest

The authors declare no conflict of interests.

## Supplementary Material

RA-009-C9RA04102H-s001
