# Supplementary material for: Dicarboxyl-terminated iron(ii) clathrochelates as ICD-reporters for globular proteins
Source: RSC Adv. 2019 Aug 5;9(42):24218–30. doi: 10.1039/c9ra04102h (PMC9069836; doi:10.1039/c9ra04102h)
Supplement: RA-009-C9RA04102H-s001 [file RA-009-C9RA04102H-s001.pdf]

Electronic Supplementary Material (ESI) for RSC Advances.
This journal is © The Royal Society of Chemistry 2019

Supporting information

for

DICARBOXYL-TERMINATED IRON(II) CLATHROCHELATES AS CD-REPORTERS FOR A SERIES OF GLOBULAR PROTEINS

VLADYSLAVA KOVALSKA[1*], SERHII VAKAROV[2,3], MYKHAYLO LOSYTSKYY[1], MARINA KUPERMAN[1], NINA CHORNENKA[2,3], YULIYA TOPORIVSKA[4], ELZBIETA GUMIENNA-KONTECKA[4], YAN VOLOSHIN[5,6], OLEG VARZATSKII[3], ANDRIY MOKHIR[7]

[1]Institute of Molecular Biology and Genetics, NASU, 150 Zabolotnogo St., 03143 Kyiv, Ukraine, e-mail: v.kovalska@gmail.com

[2] Princeton Biomolecular Research Labs, 26A Saperne pole St., 01042, Kyiv, Ukraine

[3] Institute of General and Inorganic Chemistry, 32/34 Palladin Av., 03142 Kyiv, Ukraine

[4] Faculty of Chemistry, University of Wroclaw, 14 F. Joliot-Curie St., 50-383 Wroclaw, Poland

[5] Nesmeyanov Institute of Organoelement Compounds RAS, 28 Vavilova St., 119991, Moscow, Russia

[6] Kurnakov Institute of General and Inorganic Chemistry of the Russian Academy of Sciences, 31 Leninsky Prosp., 119991 Moscow, Russia

[7] Organic Chemistry II, Friedrich-Alexander-University of Erlangen-Nuremberg, Henkestr. 42, 91054 Erlangen, Germany

1. NMR spectra

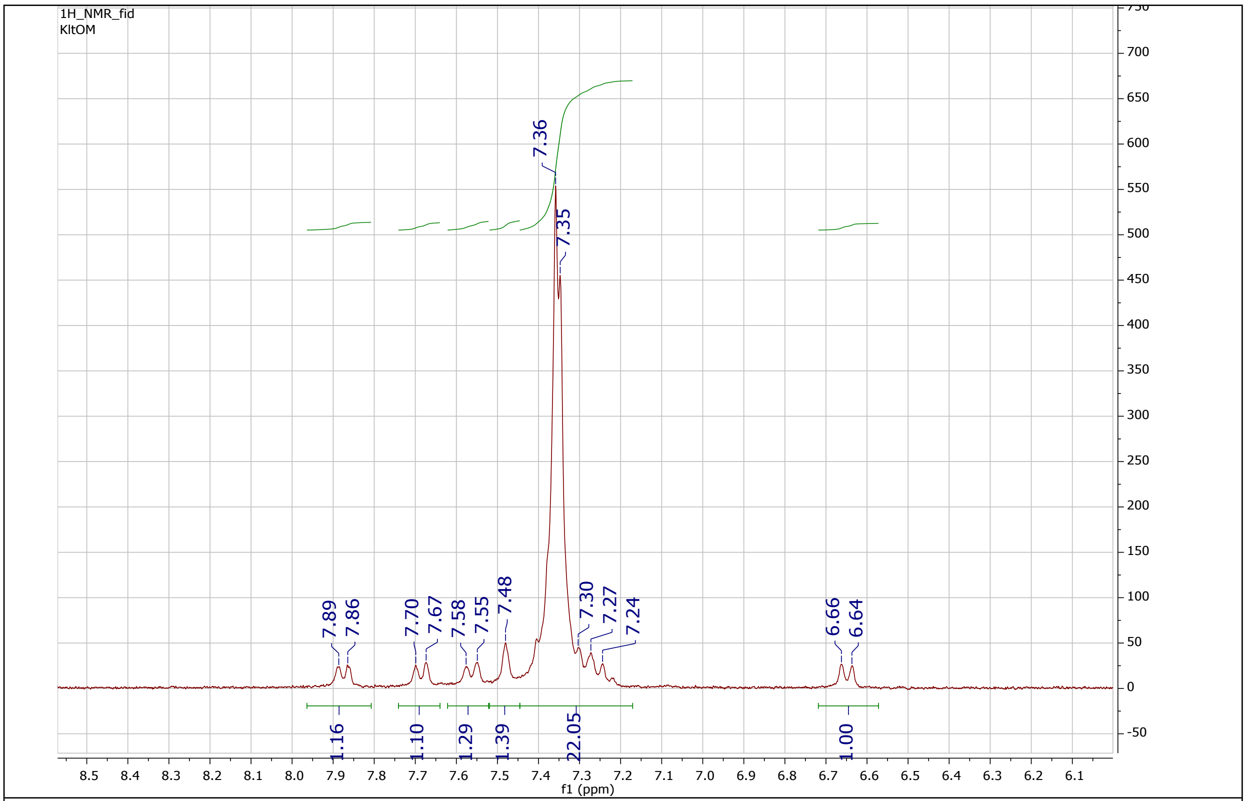

a

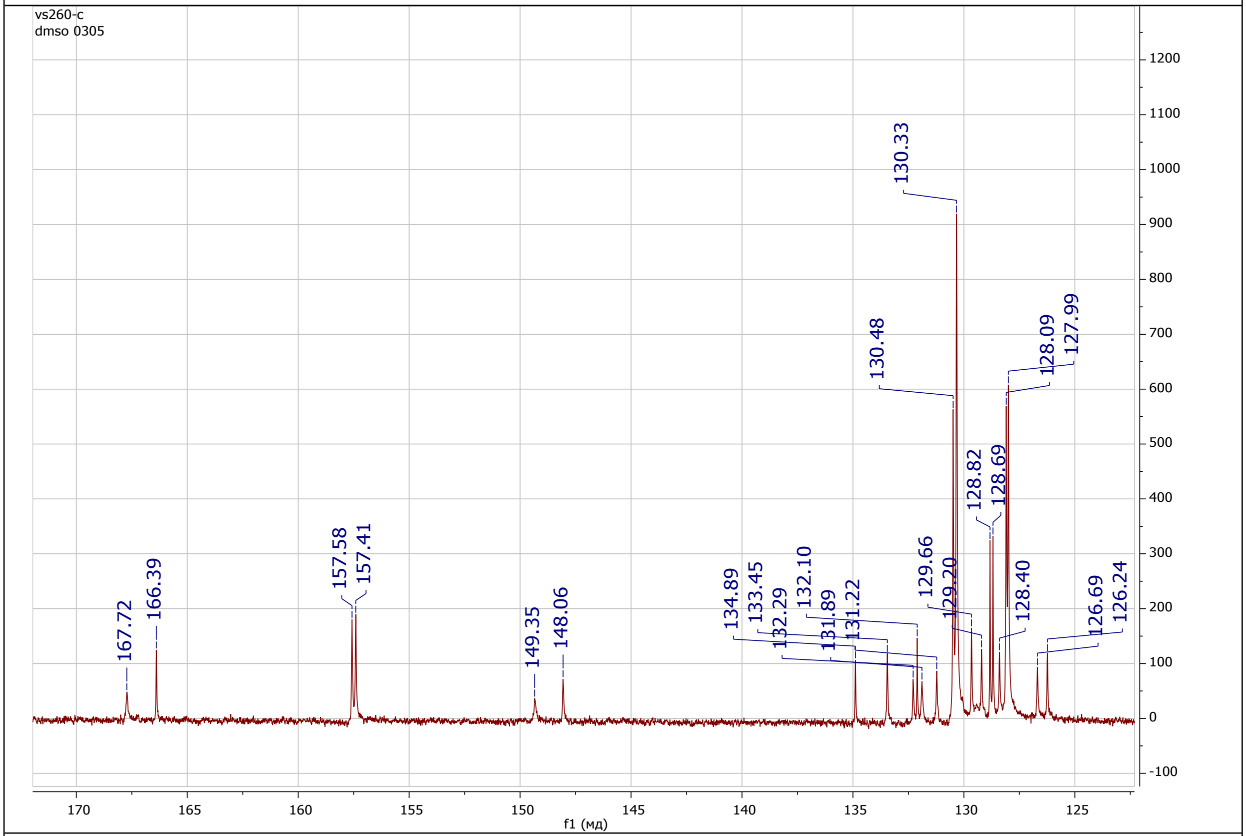

b

Figure S1. ^{1}H NMR (**a**) and ^{13}C NMR (**b**) spectra of clathrochelate ***FeBd$_2$(((meta,ortho-(HOOCC$_6$H$_4$S)$_2$Gm)(BF)$_2$*** (**4**)

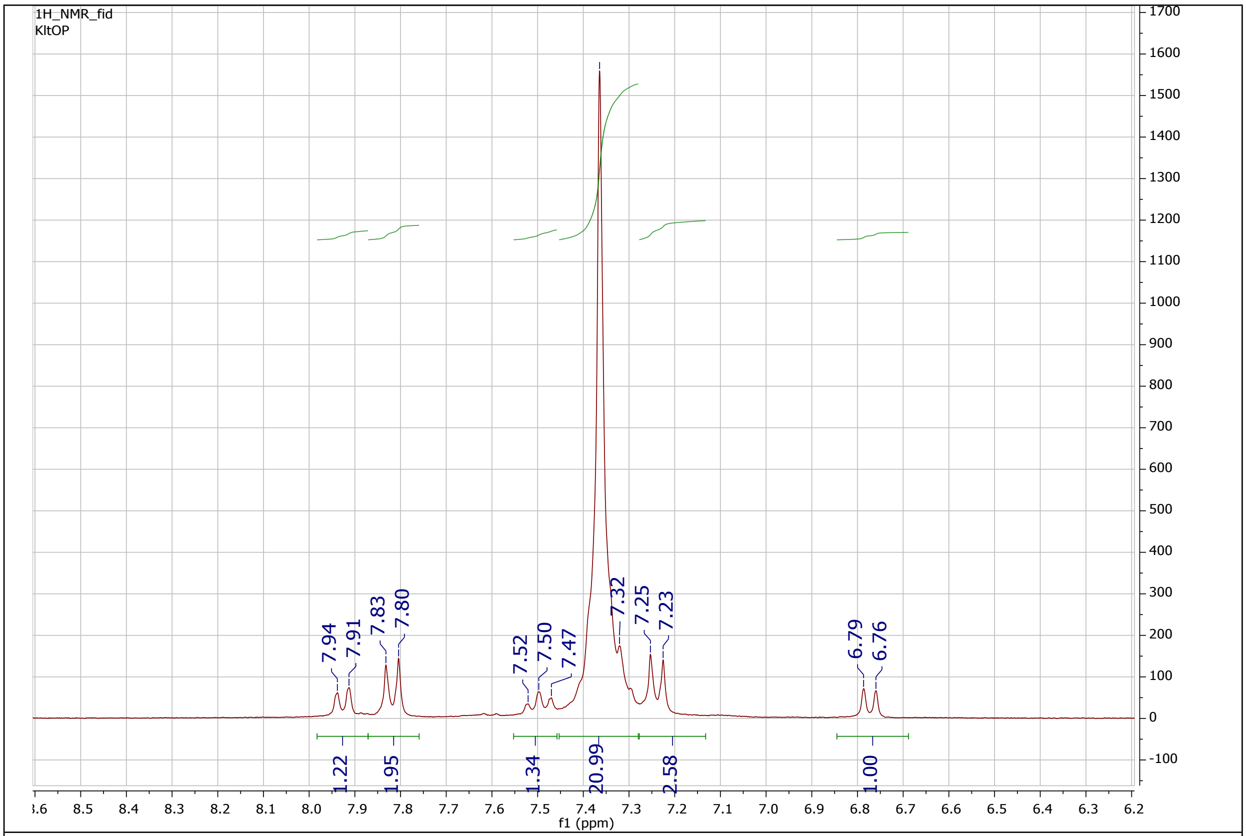

a

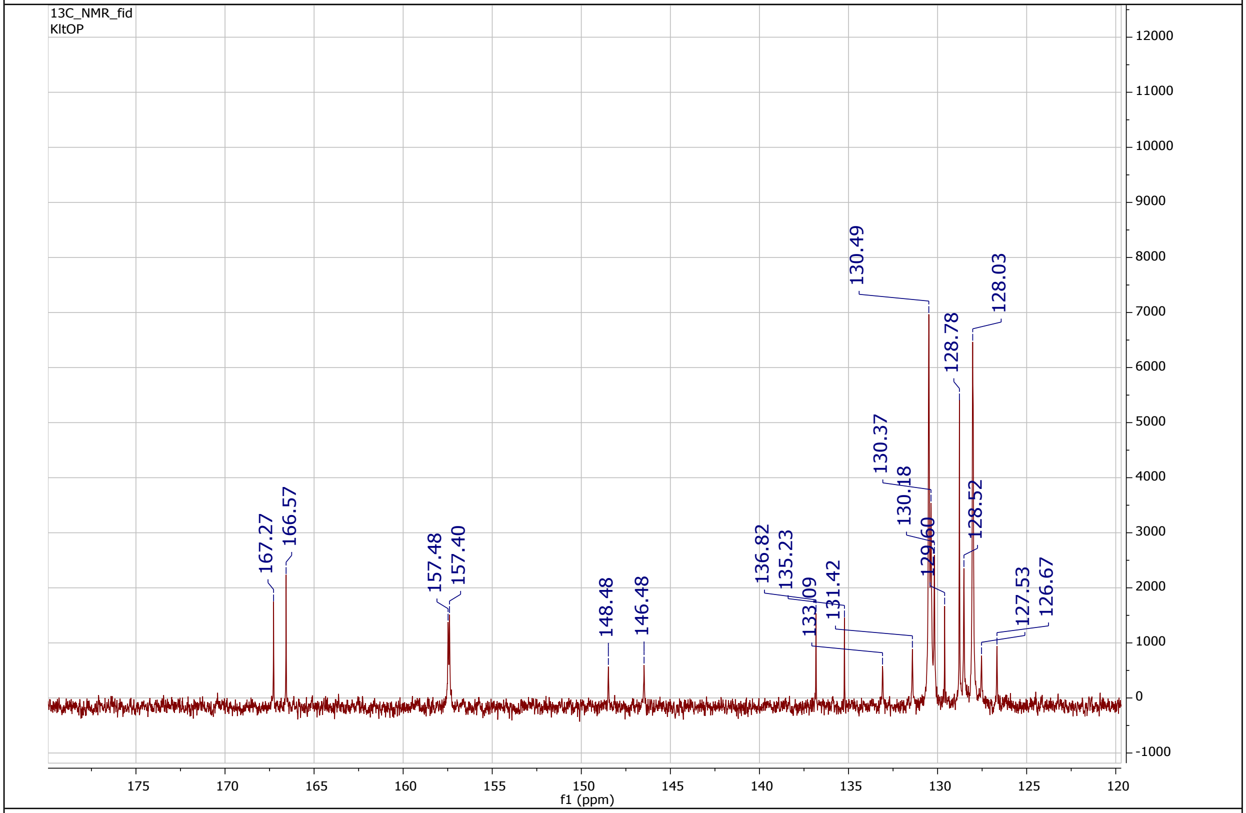

b

Figure S2. ^{1}H NMR (**a**) and ^{13}C NMR (**b**) spectra of clathrochelate ***FeBd$_2$(((para,ortho-(HOOCC$_6$H$_4$S)$_2$Gm)(BF)$_2$*** (**5**)

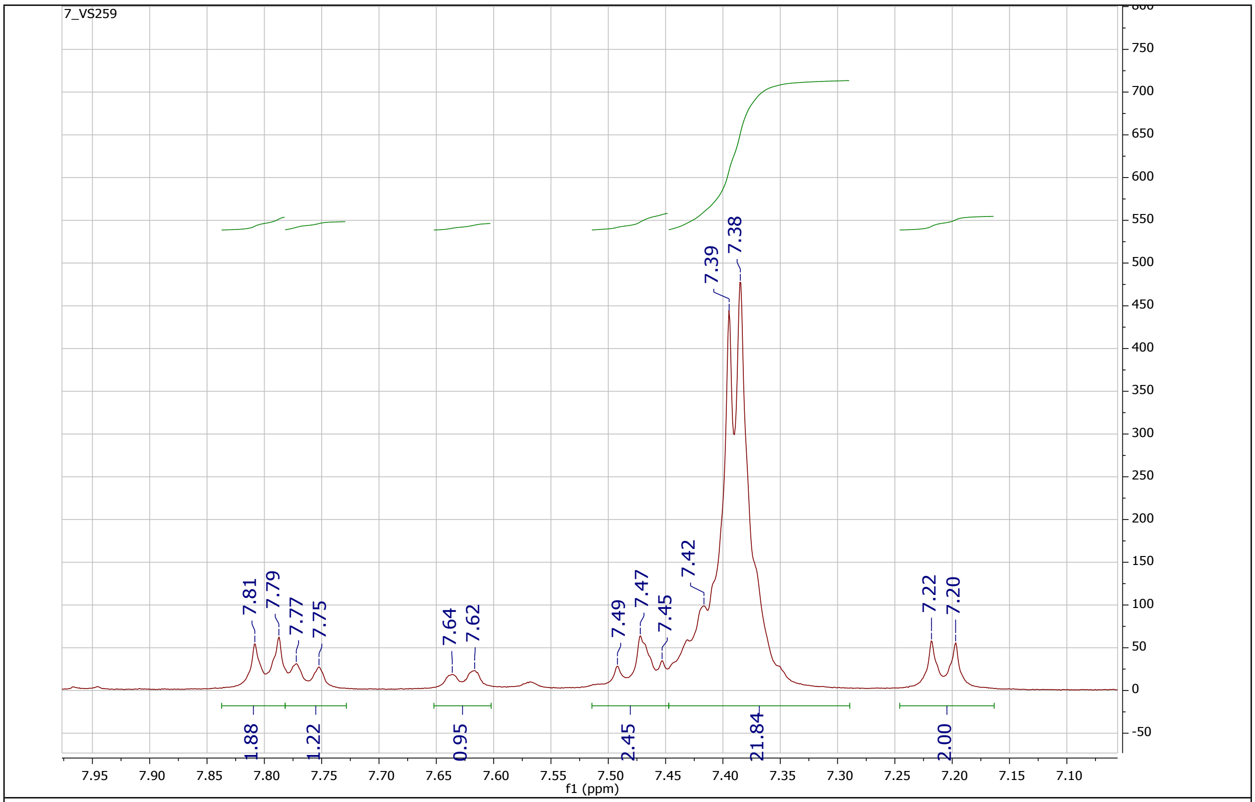

a

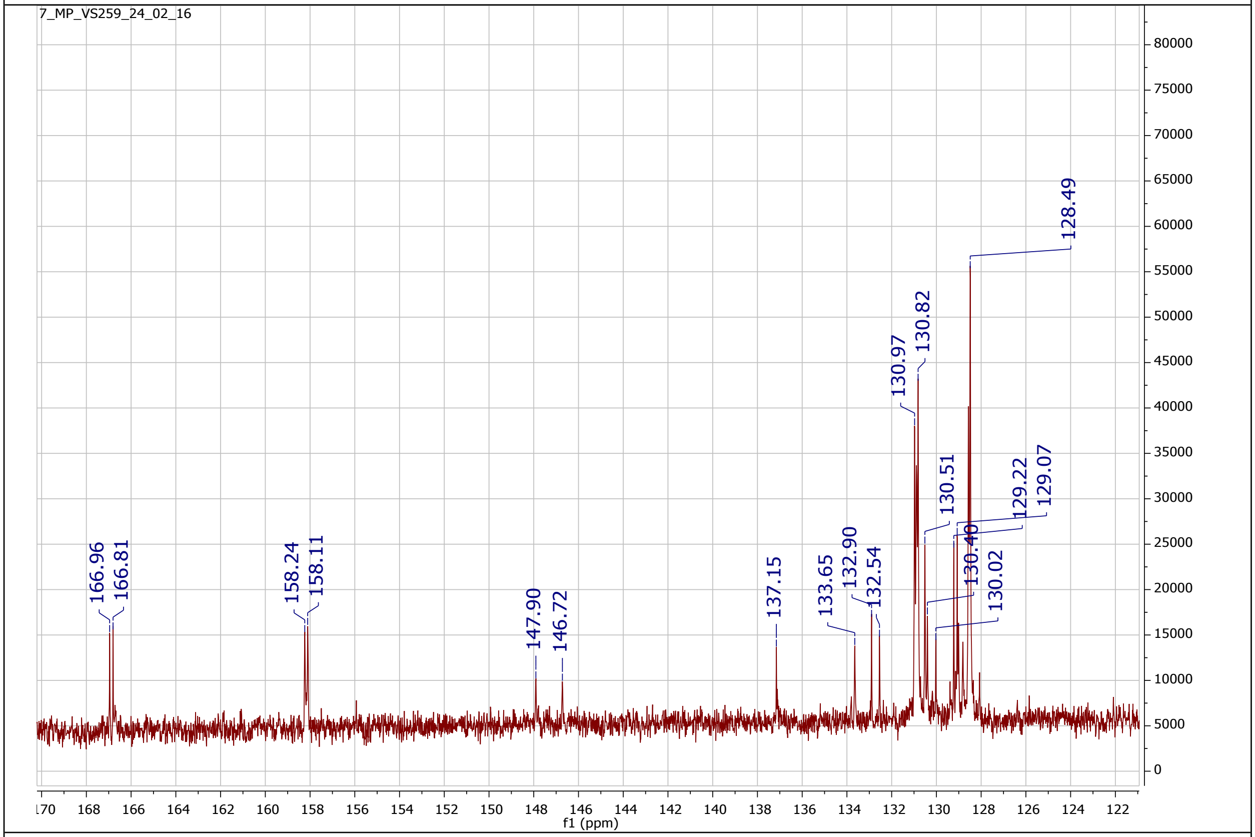

b

Figure S3. ^{1}H NMR (**a**) and ^{13}C NMR (**b**) spectra of clathochelate ***FeBd$_2$(((para,meta-(HOOCC$_6$H$_4$S)$_2$Gm)(BF)$_2$*** (**6**)

2. Protein fluorescence quenching curves

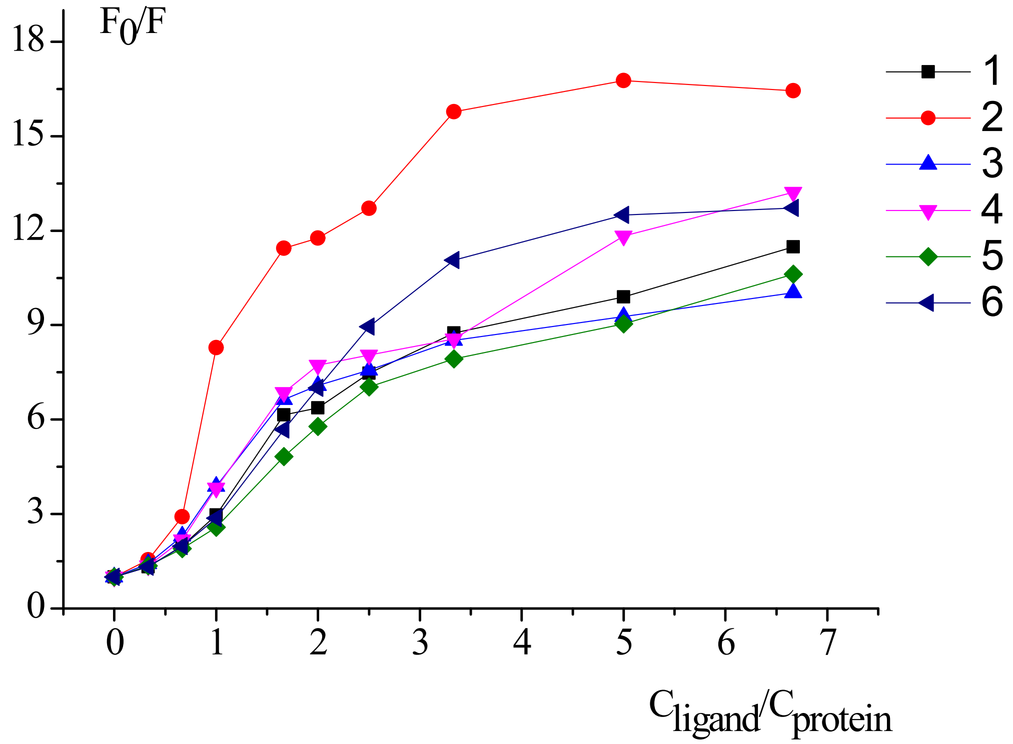

Figure S4. Stern–Volmer plots of a fluorescence quenching of **BSA** by the complexes **1** – **6**, excitation at 280 nm, emission at 344 nm, $c_{protein}$ = 3 µM, 0.05M Tris–HCl buffer with pH 7.9 used as a solvent at 25° C

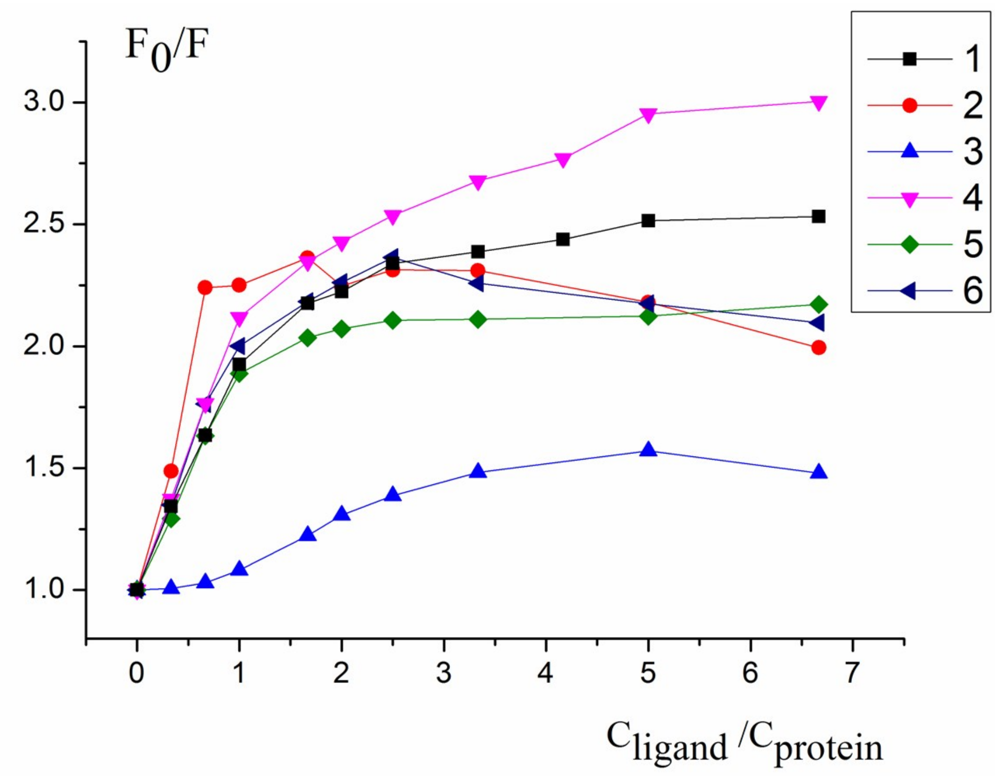

Figure S5. Stern–Volmer plots of a fluorescence quenching of **HSA** by the complexes **1** – **6**, ex= 280 nm, emission at 353 nm, $c_{protein}$ = 3 μM, 0.05M Tris–HCl buffer with pH 7.9 used as a solvent at 25° C

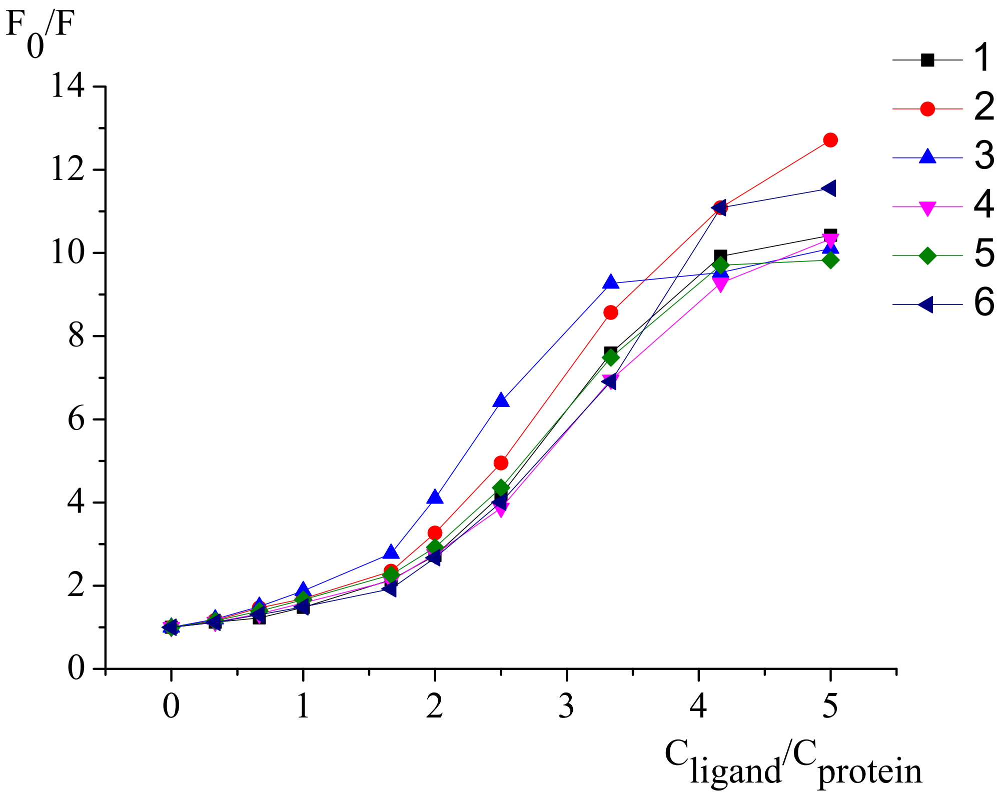

Figure S6. Stern–Volmer plots of a fluorescence quenching of **LYZ** by the complexes **1** – **6** ex=280 nm, emission at 344 nm, $c_{protein}$ = 3 μM, 0.05M Tris–HCl buffer with pH 7.9 used as a solvent at 25° C.

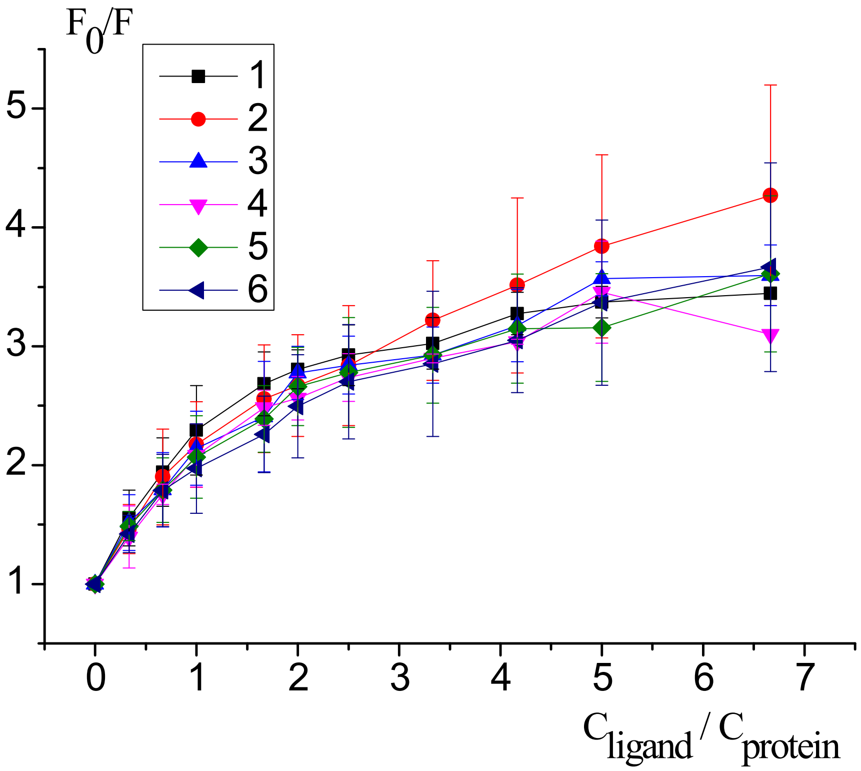

Figure S7. Stern–Volmer plots of a fluorescence quenching of **BLG** by the complexes **1** – **6**, ex=280 nm, emission at 336 nm, $c_{protein}$ = 3 µM, 0.05M Tris–HCl buffer with pH 7.9 used as a solvent at 25° C.

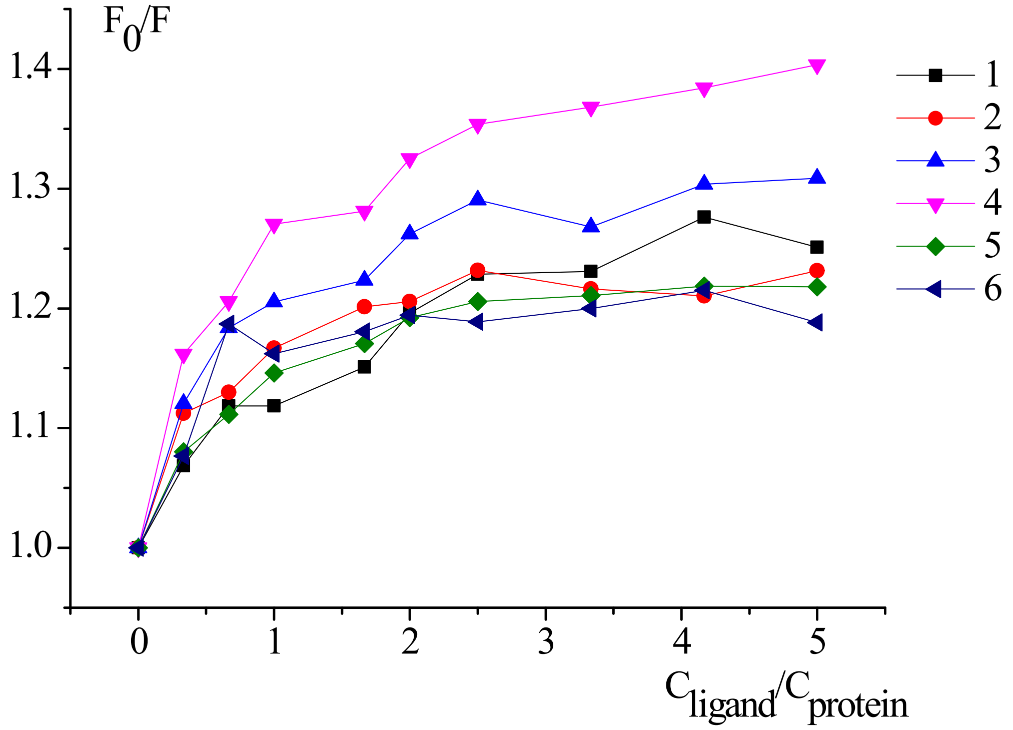

Figure S8. Stern–Volmer plots of a fluorescence quenching of **trypsin** by the complexes **1** – **6** ex=280 nm, emission at 343 nm, $c_{protein}$ = 3 μM, 0.05M Tris–HCl buffer with pH 7.9 used as a solvent at 25° C

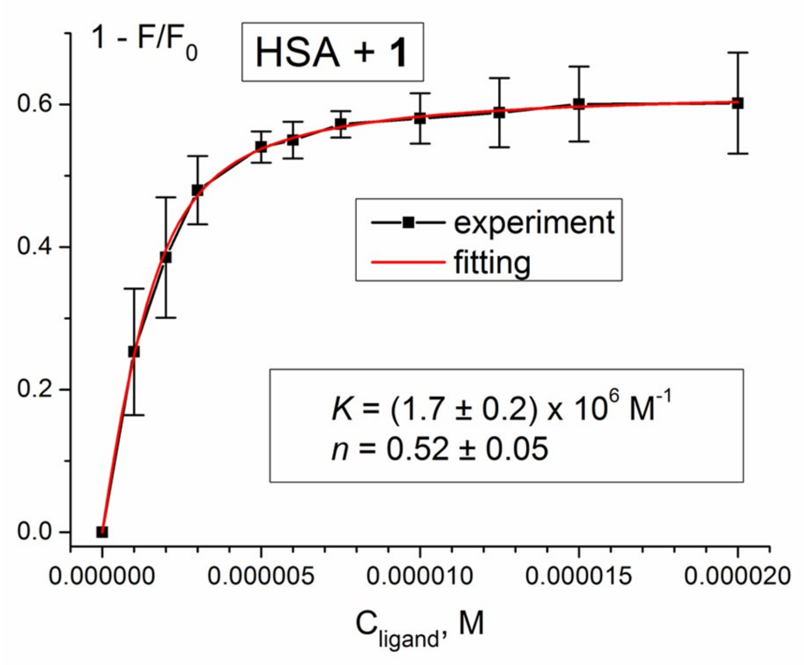

a

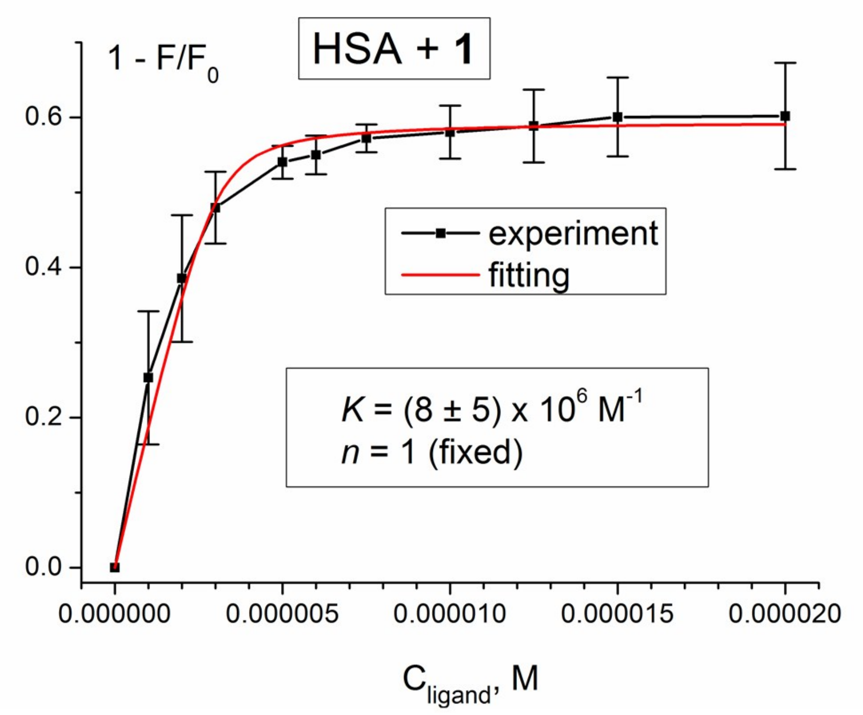

b

Fig. S9. The plots of ($1 - F/F_0$) on the concentration of the clathrochelate (C_{ligand}) for the titration of HSA (3 μM) with the compound **1**, and the fit of this plot with the dependence (5) (see below, Section 5 of the SI). 0.05M Tris–HCl buffer with pH 7.9 used as a solvent. Experiment performed three times, average values of ($1 - F/F_0$) are presented; error bars correspond to confidence level of 0.95. *K* is found as the parameter of fitting; *n* is found as the parameter of fitting (a), and is fixed as 1 (b).

a

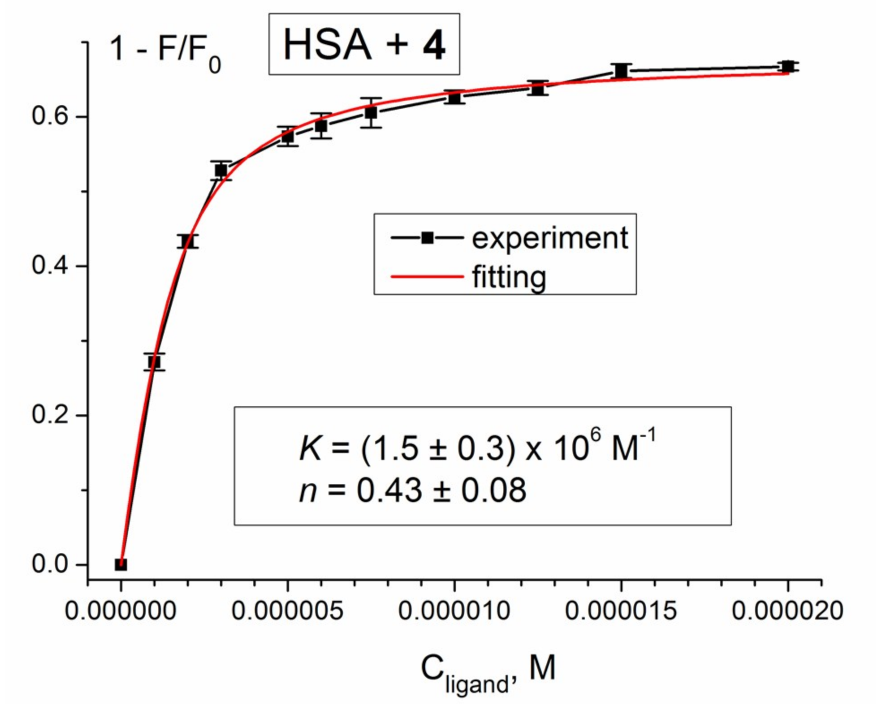

b

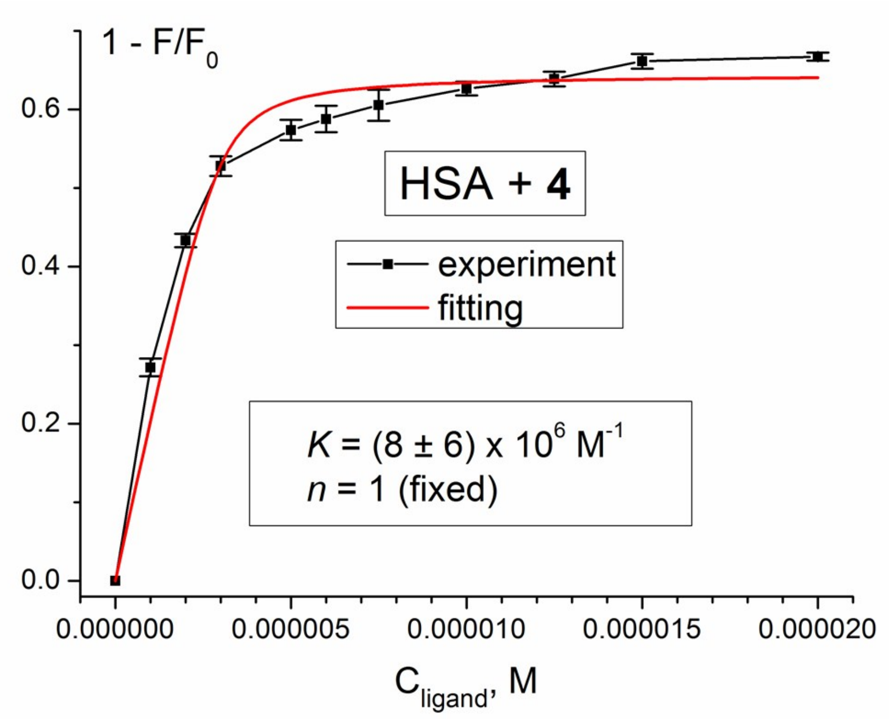

Fig. S10. The plots of $(1 – F/F_0)$ on the concentration of the clathrochelate (C_{ligand}) for the titration of HSA (3 μM) with the compound **4**, and the fit of this plot with the dependence (5) (see below, Section 5 of the SI). 0.05M Tris–HCl buffer with pH 7.9 used as a solvent. Experiment performed three times, average values of $(1 – F/F_0)$ are presented; error bars correspond to confidence level of 0.95. *K* is found as the parameter of fitting; *n* is found as the parameter of fitting (a), and is fixed as 1 (b).

3. Fluorescence spectra

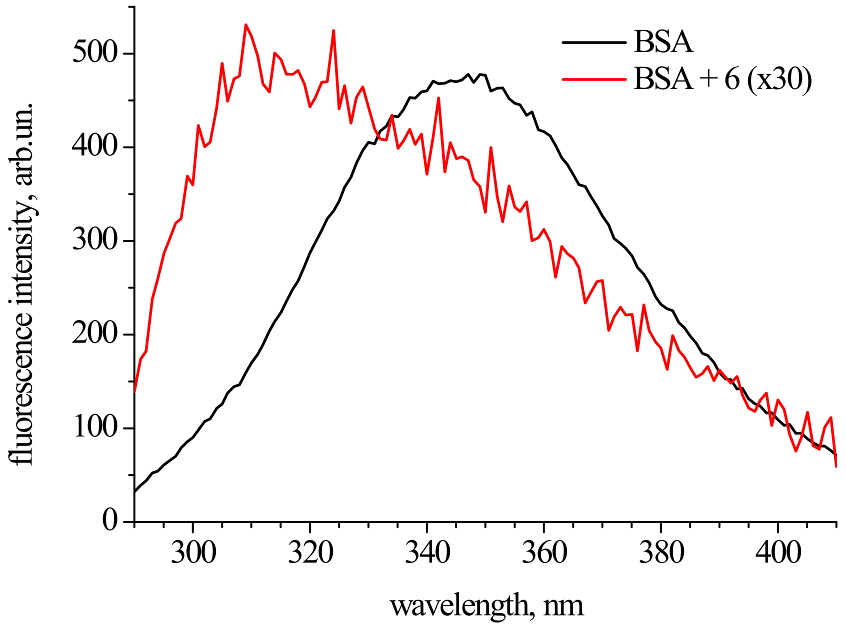

a

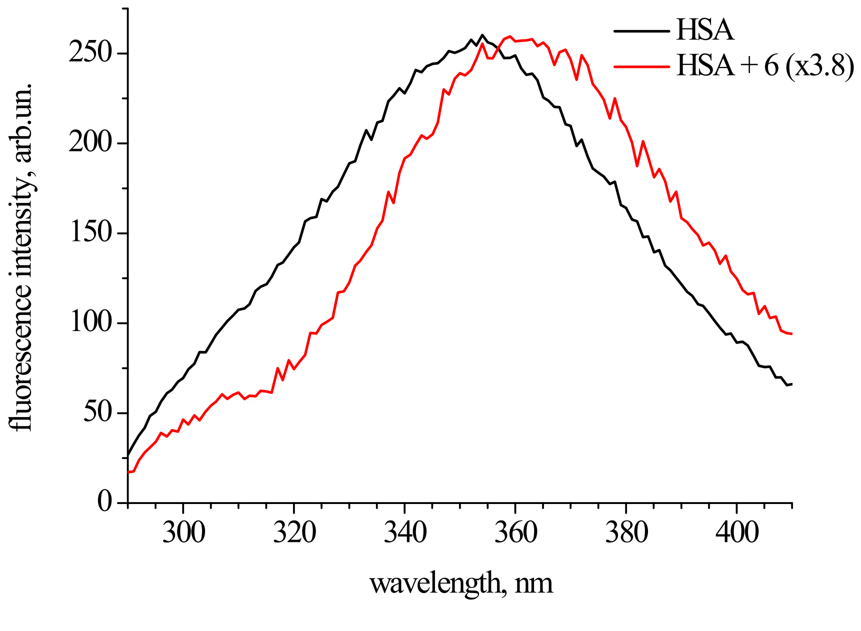

b

Figure S11. Fluorescence spectra of BSA at excitation wavelength of 280 nm (**a**) and of HSA (**b**) in the absence and in the presence of the clathrochelate **6**. $c_{protein} = 3 \cdot 10^{-5}$ mol·l^{-1}, $c_{clt} = 10 \cdot 10^{-5}$ mol·l^{-1}, 0.05M Tris-HCl buffer with pH 7.9. The spectra quenched by **6** were normalized to the initial, non-quenched, spectra.

4. CD spectra

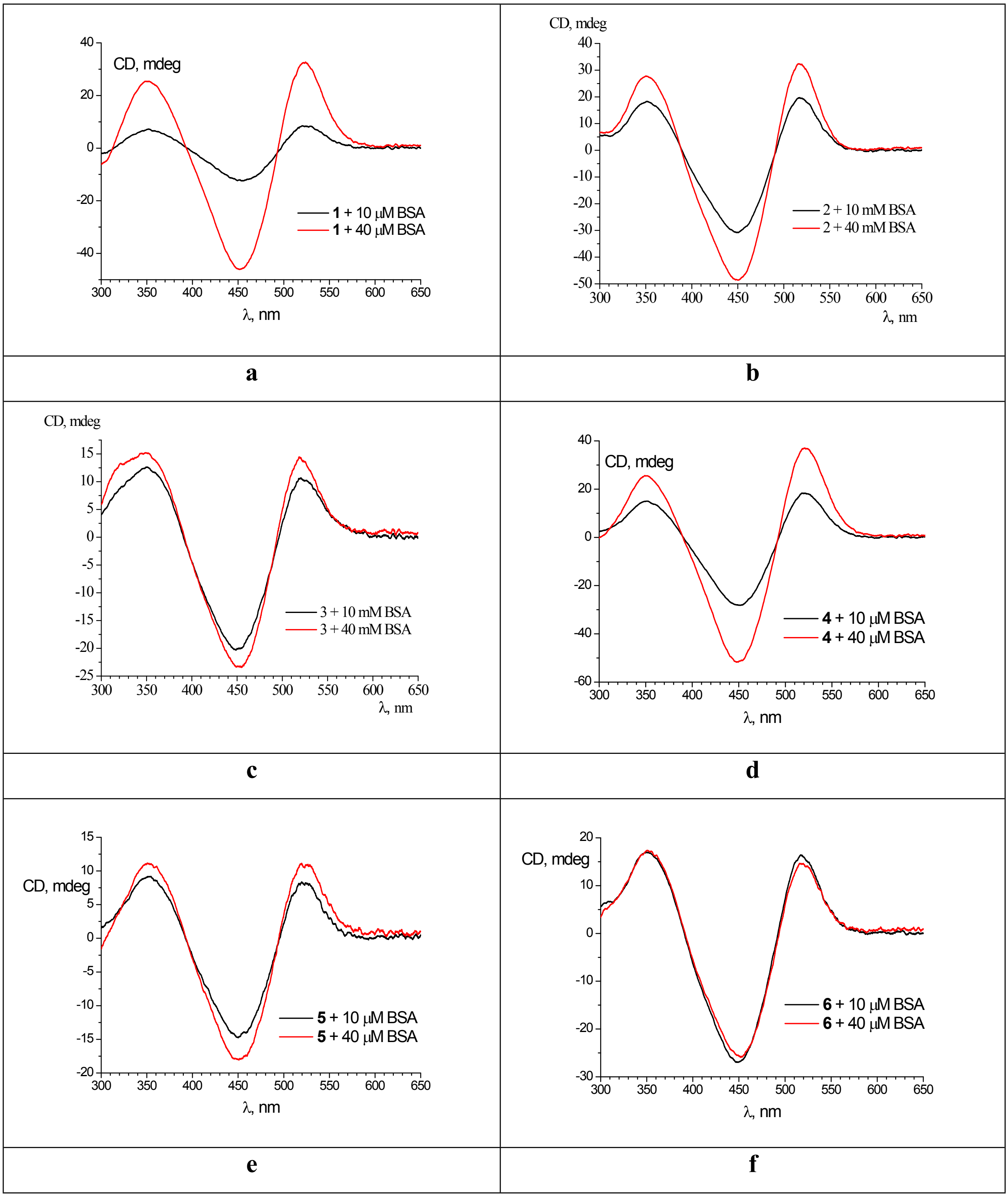

Figure S12. CD spectra of the protein – clathrochelate assemblies of BSA with clathrochelates **1** (**a**), **2** (**b**), **3** (**c**), **4** (**d**), **5** (**e**), and **6** (**f**) which are recorded at $c_{protein} = 4\cdot10^{-5}$ and $1\cdot10^{-5}$ mol·l^{-1}, $c_{clt} = 2\cdot10^{-5}$ mol·l^{-1} in 0.05M Tris-HCl buffer with pH 7.9.

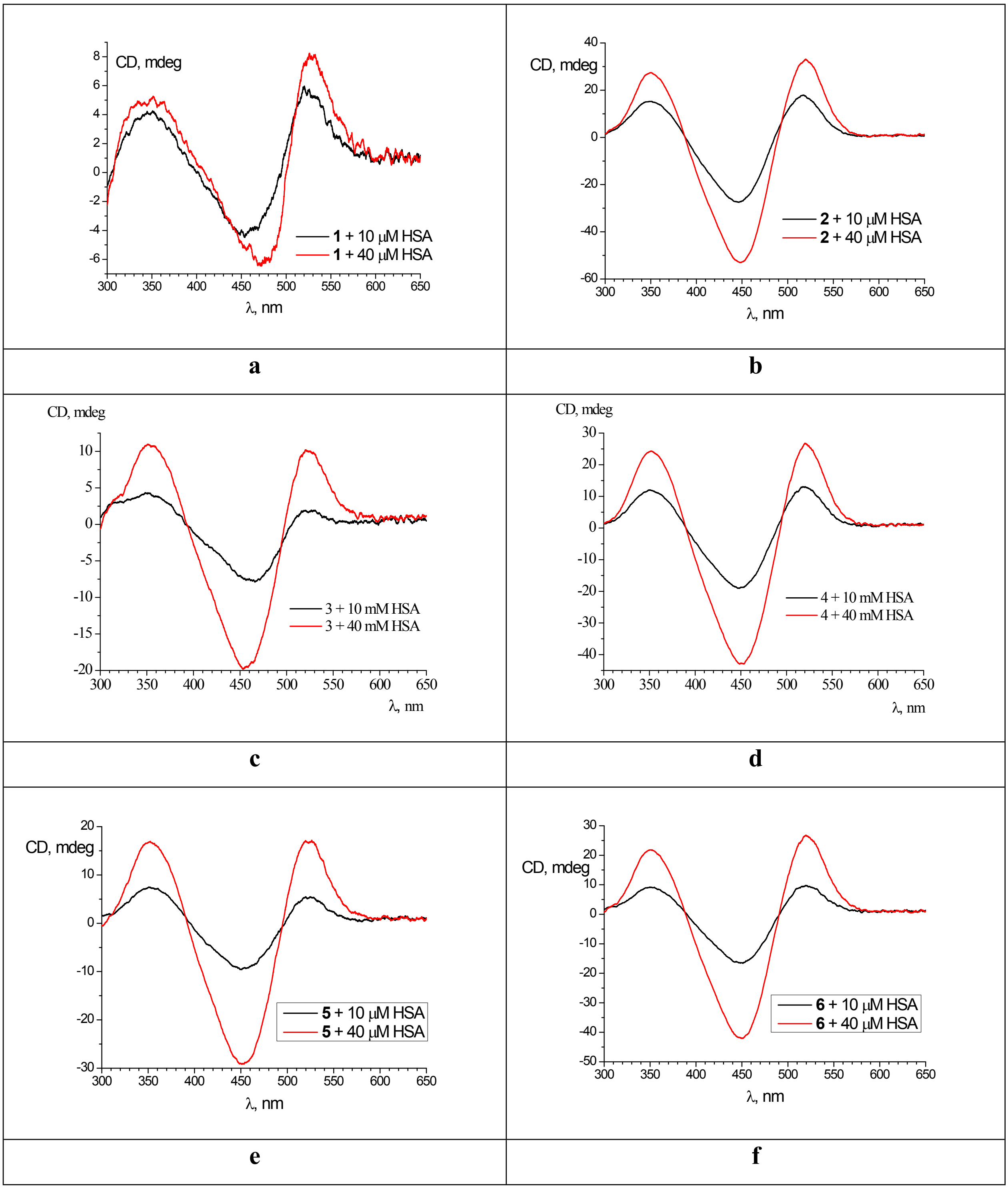

Figure S13. CD spectra of the protein – clathrochelate assemblies of HSA with clathrochelates **1** (**a**), **2** (**b**), **3** (**c**), **4** (**d**), **5** (**e**), and **6** (**f**) which are recorded at $c_{protein} = 4 \cdot 10^{-5}$ and $1 \cdot 10^{-5}$ mol·l^{-1}, $c_{clt} = 2 \cdot 10^{-5}$ mol·l^{-1} in 0.05M Tris-HCl buffer with pH 7.9.

5. Estimation of affinity and stoichiometry of clathrochelates binding to HSA

For HSA that contains single Trp residue, we expected the relation of fluorescence quenching curve to binding isotherm to be less complicated as compared to other proteins studied (containing multiple Trp residues). Hence, binding stoichiometry and binding affinity were estimated for the studied clathrochelates; for those where the fitting was the most adequate, the values were provided. Starting with the assumption that each protein globule has *n* sites for clathrochelate binding with the equal values of the binding constant *K*, the following expression could be written:

$$K = \frac{C_{BL}}{(C_L - C_{BL}) \times (n \times C_P - C_{BL})} \qquad (1)$$

where *K* is the binding constant; *n* is the number of clathrochelate molecules bound per HSA globule; C_P, C_{BL} and C_L are the concentrations of protein globules, HSA-bound ligand (i.e. clathrochelate) molecules and total ligand concentration respectively. Solving the quadratic equation, we obtain:

$$C_{BL} = \frac{n \times C_P}{2} + \frac{C_L}{2} + \frac{1}{2 \times K} - \sqrt{\left(\frac{n \times C_P}{2} + \frac{C_L}{2} + \frac{1}{2 \times K}\right)^2 - n \times C_P \times C_L} \qquad (2)$$

To obtain the relation between C_{BL} and the observed fluorescence intensity of the protein (*F*), we supposed that the protein fluorescence intensity is proportional to the number of non-occupied binding sites. In this case, the observed fluorescence intensity of the protein could be expressed as:

$$F = \frac{n?—C_P - C_{BL}}{n?—C_P} ?—F_0 + \frac{C_{BL}}{n?—C_P} ?—F_{min} \qquad (3)$$

where F_0 and F_{min} are fluorescence intensities of the protein in the absence of clathrochelates, and protein with all binding sites occupied with clathrochelate molecules, respectively. It could be obtained from (3):

$$\frac{C_{BL}}{n?—C_P} = \frac{F_0 - F}{F_0 - F_{min}} \qquad (4)$$

Together, (2) and (4) result in the final equation:

$$Y = A \times \left[\frac{1}{2} + \frac{x}{2 \times C_P \times n} + \frac{1}{2 \times K \times C_P \times n} - \sqrt{\left(\frac{1}{2} + \frac{x}{2 \times C_P \times n} + \frac{1}{2 \times K \times C_P \times n}\right)^2 - \frac{x}{C_P \times n}}\right] \qquad (5)$$

where $x = C_L$, $Y = (1 – F/F_0)$, $A = (1 – F_{min}/F_0)$. Thus, the experimentally obtained curve of protein fluorescence quenching is presented as the dependence of $(1 – F/F_0)$ on C_L, and further fitted with the dependence (5). As a result of this fitting, the values of K, n and A are obtained as fitting parameters.
